# The enhanced dewaterability of sludge by a starch-based flocculant combined with attapulgite

**DOI:** 10.1038/s41598-023-27749-3

**Published:** 2023-01-09

**Authors:** Shaohang Shen, Hua Wei, Yu Pan, Pan Hu, Hu Yang

**Affiliations:** 1 State Key Laboratory of Pollution Control and Resource Reuse, School of the Environment, Nanjing University, Nanjing, 210023 People’s Republic of China; 2 School of Ecology Technology and Engineering, Shanghai Institute of Technology, Shanghai, 201418 People’s Republic of China

**Keywords:** Environmental sciences, Environmental chemistry

## Abstract

Coagulation/flocculation is one of the most widely used and cost-effective pretreatment methods for improving the dewaterability of sludge. In this study, a cationic modified starch-based flocculant (St-CTA) in conjunction with a popular clay, attapulgite (ATP), was used for the conditioning of waste-activated sludge. The dewatering properties, including the filter cake moisture content, filtration specific resistance, capillary suction time, filtration rate and compressibility coefficient, were measured and compared by varying the doses of St-CTA and ATP. By combination of the apparent dewatering performance and the changes in the contents and distributions of the extracellular polymeric substance (EPS) fractions and components, sludge flocs, and microstructures of sludge cakes, the dewatering mechanisms were discussed in detail. St-CTA in conjunction with ATP can exhibit an enhanced dewaterability of sludge and the water content in final sludge cake can be stably reduced below 80% owing to the synergistic effects of St-CTA and ATP. In addition to the efficient charge neutralization of St-CTA, ATP not only acts as a skeleton builder in the sludge dewatering process which makes the sludge flocs more compact and improves the filterability and permeability, but also tightly interacts with the proteins in EPS of the sludge which reduces the protein content and further enhances the dewatering effect. This study provides an economical, green, and effective way to further improve the dewaterability of sludge.

## Introduction

In recent years, with the steady improvement of the world economy, the scale of water and wastewater treatment have notably increased, and accordingly the discharge of sludge as a by-product of wastewater treatment has consistent growth^1^. However, raw sludge usually contains more than 95% water^2^, the high volume of which causes its high transport and disposal costs^3^. It is therefore vitally important to reduce the volume of sludge by the dewatering processes for its efficient treatment^4^.

Sludge is usually conditioned by some pre-treatments before the mechanical squeezing to efficiently separate water from the sludge^5^. Sludge pre-treatments, mainly including coagulation/flocculation, oxidation, acidification, skeleton building, microwave and ultrasonic conditioning, can convert the surface adsorbed water and internal hydrated water into free water, in which coagulation/flocculation is a widely used one in the wastewater treatment plants because of its simplicity and effectiveness^6^. Traditional coagulants and flocculants, such as polyaluminium chloride, polyferric sulfate and cationic polyacrylamide (PAM), were widely used because they can effectively aggregate the sludge particles and achieve the solid–liquid separation^6^. However, the residual metal ions and highly toxic monomers of these traditional coagulants and flocculants may cause the potential environmental risks^7^. Moreover, the traditional coagulation/flocculation is difficult to achieve a complete dewatering because the highly hydrated organic matters in sludge such as the extracellular polymeric substance (EPS), binding large amounts of water, were difficult to be destroyed to fully release the bound and intracellular water^8,9^. Besides, highly compressible sludge cakes would compact fully under the high mechanical pressure, causing the drainage channels destroyed, the filterability reduced and the internal bound water prevented from being completely drained^6,10,11^. Therefore, coagulation/flocculation used solo is difficult to meet the high requirements of sludge dewatering, and an environmentally-friendly and effective combined conditioning processes with other pre-treatments can notably improve the dewaterability of sludge^1,6,12,13^.

Commonly combined conditioning processes included acidification-coagulation^12^, oxidation-coagulation^13^, and coagulation and skeleton builder combination process^1^. Among them, acidification-coagulation and oxidation-coagulation can effectively destroy the EPS and release the intracellular water but cannot enhance the filterability of sludge cakes^14^. The coagulation and skeleton builder combination process is based on the coagulation and the addition of some skeleton builders, as filter aids, to condition the sludge^5^. Some skeleton builders, such as bentonite, fly ash and biomass ash, are widely available and inexpensive, besides, contain high levels of SiO_2_ with a good mechanical strength^4,15–17^. With the support of the skeleton builders, the sludge cake contains rigid and porous microstructure even under a high mechanical pressure, reducing the compressibility of sludge, allowing the water contained in the sludge cakes to drain out easily, and thus improving the permeability and mechanical strength of sludge to achieve a high solid content in sludge (> 30% dry sludge volume)^4,15–17^. The coagulation and skeleton builder combination process thus has the advantages of high-cost performance and ease of operation by the direct addition of flocculants and skeleton builders without any adjustment^6,7^; besides, the porous and rigid structures of the skeleton builders can easily change the sludge compressibility and further enhance the sludge dewatering performance by this combining process.

Starch is one of the environmentally-friendly and low-cost polysaccharides^18^. In this work, an etherified cationic starch-based flocculant with a high charge density, namely starch-3-chloro-2-hydroxypropyltrimethylammonium chloride (St-CTA), was synthesized^19^, on which the quaternary ammonium cationic groups can also effectively damage the cellular structures and interact with EPS. St-CTA has thus shown a notable effect on sludge dewatering according to previous reports^13,19^. Besides, some clays, such as montmorillonite and kaolin, have been confirmed as efficient skeleton builders in combination with various flocculants for effective conditioning of sludge^17,20^. Attapulgite (ATP), a popular clay in China, has the characteristics of the high specific surface area, the rigid and porous structure and good adsorption properties to many organic matters^21,22^. ATP, as a skeleton builder, was thus in conjunction with St-CTA here. The dewatering performance of their combined usages for waste-activated sludge with different dosing sequences and various dosing ratios was assessed in terms of specific resistance of filtration (SRF), filter cake moisture content (FCMC), time to filter (TTF), floc properties and microstructures of sludge cakes. Changes in the EPS distribution and composition of the conditioned sludge were investigated relative to the final dewatering efficiency. The synergistic mechanisms of St-CTA and ATP were also discussed in detail. This work thus proposes a novel economical combination conditioning method for sludge dewatering.

## Results and discussion

### Sludge dewatering performance

#### Dosing sequences

Before the dewatering experiment, the effects of three different dosing sequences using grinded or not grinded ATP on the sludge dewatering, i.e. ATP dosed before, together, or after St-CTA, were studied and compared firstly, as shown in Supporting Information Figs. S1, S2. According to Fig. S1, the FCMC and SRF of sludge after conditioned by this combination had no evident differences under the three different dosing sequences. However, St-CTA dosed before ATP showed slightly better dewatering performance with increasing ATP dose, by which the optimal FCMC and SRF were around 79.50% and 0.30 × 10^12^ m/kg, respectively, at the ATP dose approximately 10.00 kg/m^3^ (Fig. S1e–f). This finding might be due to that the ATP could easily combine with the primary sludge flocs formed by the previously fed St-CTA to improve the filterability and permeability of the final sludge cakes. Similarly, Supporting Information Fig. S2 shows no notable change in the FCMC and SRF of sludge by using grinded or not grinded ATP with the average particles size approximately 8.577 μm and 18.107 μm respectively (Fig. S2g). However, a slight better dewatering performance were obtained using not grinded ATP when the ATP dose increased to 10.00 kg/m^3^ (Fig. S2e–f). Accordingly, the conditioning process of unground ATP fed after St-CTA was highly efficient and convenient, which was thus applied in the following dewatering experiments.

#### Dose of St-CTA

**Figure 1:**
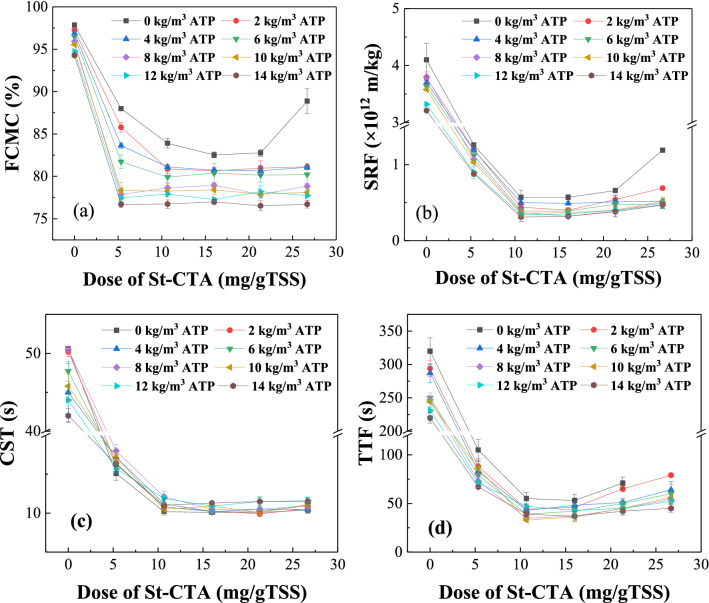
The sludge dewatering performance of St-CTA in conjunction with ATP by using different doses: (**a**) FCMC, (**b**) SRF, (**c**) CST and (**d**) TTF.

**Figure 2:**
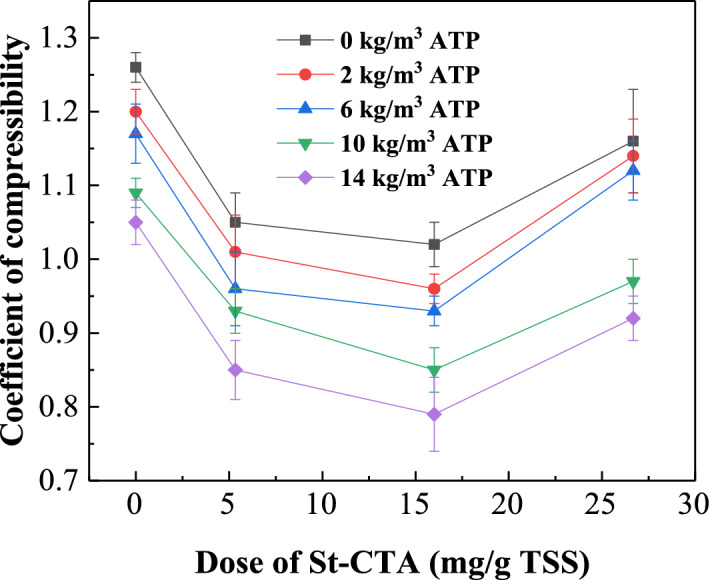
The compressibility coefficients of sludge conditioned by St-CTA in conjunction with ATP using different doses.

**Figure 3:**
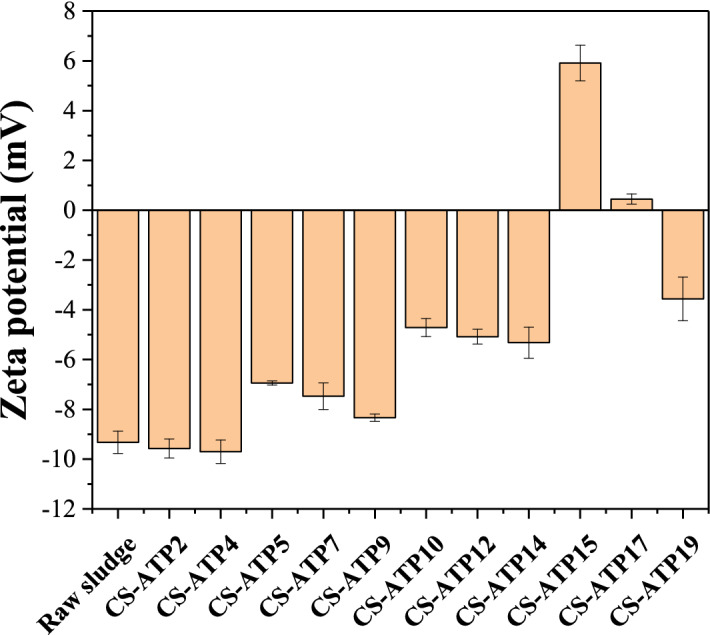
The zeta potentials of the sludge supernatants conditioned by St-CTA in conjunction with ATP using different doses.

The various conditioning processes with different doses of St-CTA and ATP, named CS-ATP1—CS-ATP19, were conducted to sludge and their dewatering performance are shown in Figs. 1,2, Table 1, and Supporting Information Figs. S3–S5. According to Figs. 1,2, Table 1, and Supporting Information Figs. S3–S5, the dewatering performance of sludge conditioned with St-CTA individually evidently improved especially at the low dose range but reached a plateau when St-CTA was dosed at the optimal dose of 16.00 mg/gTSS or even deteriorated after the optimal doses, specifically, the FCMC reduced from 97.86 to 82.52%, SRF was from 4.10 × 10^12^ to 0.57 × 10^12^ m/kg, CST was from 50.6 to 10.1 s, TTF was from 320 to 53 s, the compressibility coefficients was from 1.26 to 1.02, the filtrate volume was from 32.1 to 95.0 mL and the filtration rate was from 0.2546 to 1.4218 m^3^/(m^2^ h) at the optimal dose. This finding confirmed that the charge naturalization of St-CTA was crucial to the sludge conditioning process and the excessive positive charges might lead to the restabilization of sludge colloidal dispersions (CS-ATP5, CS-ATP10 and CS-ATP15, Fig. 3)^6,23,24^. Besides, St-CTA, a polysaccharide-based material with a rigid chain structure, could still act as a skeletal builder to build drainage channels, which was conducive to water run-off and thus improving the sludge dewatering^6^. Based on Figs. 1,2, Table 1, and Supporting Information Figs. S3–S5, the dewatering performance of sludge conditioned with St-CTA in conjunction with various constant doses of ATP was all improved and showed similar patterns to that with only St-CTA because of the synergistic effects of St-CTA and ATP.

**Table 1:** Sludge dewatering performance of St-CTA in conjunction with ATP by using different doses (the samples colored in bold in this table would be applied in the following experiments).

Conditioning process	Dose of St-CTA (mg/gTSS)	Dose of ATP (kg/m^3^)	FCMC (%)	SRF (× 10^12^ m/kg)	CST (s)	*s*	TTF (s)	*q*_max_ [m^3^/(m^2^·h)]
**Raw sludge**	**0.00**	**0**	**97.86 ± 0.02**	**4.10 ± 0.29**	**50.6 ± 0.2**	**1.26 ± 0.02**	**320 ± 19**	**0.2546**
CS-ATP1	0.00	2	97.27 ± 0.20	3.78 ± 0.11	50.2 ± 0.6	1.20 ± 0.03	294 ± 12	0.4315
**CS-ATP2**	**0.00**	**6**	**96.49 ± 0.03**	**3.64 ± 0.02**	**47.7 ± 1.3**	**1.17 ± 0.04**	**250 ± 1**	**0.4456**
CS-ATP3	0.00	10	95.54 ± 0.14	3.58 ± 0.01	45.8 ± 1.9	1.09 ± 0.02	244 ± 8	0.4669
**CS-ATP4**	**0.00**	**14**	**94.28 ± 0.24**	**3.21 ± 0.01**	**42.0 ± 0.9**	**1.05 ± 0.03**	**220 ± 8**	**0.4881**
**CS-ATP5**	**5.33**	**0**	**87.99 ± 0.22**	**1.26 ± 0.01**	**15.1 ± 0.9**	**1.05 ± 0.04**	**105 ± 11**	**1.0186**
CS-ATP6	5.33	2	85.79 ± 0.59	1.20 ± 0.06	16.4 ± 0.8	1.01 ± 0.05	88 ± 8	1.1035
**CS-ATP7**	**5.33**	**6**	**81.73 ± 0.81**	**1.12 ± 0.07**	**16.3 ± 0.5**	**0.96 ± 0.05**	**79 ± 6**	**1.1565**
CS-ATP8	5.33	10	78.33 ± 0.92	1.03 ± 0.13	17.2 ± 0.8	0.93 ± 0.03	71 ± 1	1.1176
**CS-ATP9**	**5.33**	**14**	**76.70 ± 0.36**	**0.88 ± 0.04**	**16.3 ± 0.9**	**0.85 ± 0.04**	**67 ± 1**	**1.3157**
**CS-ATP10**	**16.00**	**0**	**82.52 ± 0.34**	**0.57 ± 0.03**	**10.1 ± 0.2**	**1.02 ± 0.03**	**53 ± 7**	**1.4218**
CS-ATP11	16.00	2	80.65 ± 0.39	0.40 ± 0.02	10.3 ± 0.4	0.96 ± 0.02	46 ± 4	1.5986
**CS-ATP12**	**16.00**	**6**	**80.38 ± 1.12**	**0.39 ± 0.03**	**10.2 ± 0.4**	**0.93 ± 0.02**	**42 ± 7**	**1.8108**
CS-ATP13	16.00	10	78.40 ± 0.55	0.37 ± 0.07	10.9 ± 0.3	0.85 ± 0.03	36 ± 5	1.8957
**CS-ATP14**	**16.00**	**14**	**76.99 ± 0.32**	**0.32 ± 0.02**	**11.3 ± 0.2**	**0.79 ± 0.05**	**37 ± 5**	**1.8391**
**CS-ATP15**	**26.67**	**0**	**88.88 ± 1.46**	**1.19 ± 0.00**	**10.4 ± 0.4**	**1.16 ± 0.07**	**125 ± 7**	**0.8276**
CS-ATP16	26.67	2	81.14 ± 0.37	0.69 ± 0.03	10.6 ± 0.3	1.14 ± 0.05	79 ± 2	1.0752
**CS-ATP17**	**26.67**	**6**	**80.21 ± 1.07**	**0.47 ± 0.05**	**11.1 ± 0.9**	**1.12 ± 0.04**	**60 ± 10**	**1.3015**
CS-ATP18	26.67	10	78.10 ± 0.41	0.52 ± 0.05	11.0 ± 0.1	0.97 ± 0.03	56 ± 5	1.4289
**CS-ATP19**	**26.67**	**14**	**76.71 ± 0.44**	**0.47 ± 0.04**	**11.5 ± 0.4**	**0.92 ± 0.03**	**45 ± 4**	**1.6411**

#### Dose of ATP

Figures 1,2, Table 1, and Supporting Information Figs. S3–S5 show that the conditioning effect of ATP used only was quite limited (CS-ATP1–CS-ATP4) owing to ATP with a negatively surface charge (Fig. 3), which was consistent with the previous reports^1,4^. However, ATP dosed after St-CTA could further improve the dewatering performance of St-CTA, specifically, the FCMC reduced from 82.52 to 76.99% and the compressibility coefficients was from 1.02 to 0.79 conditioned by 16.00 mg/g TSS of St-CTA in conjunction with an optimal ATP dose of 14.00 kg/m^3^ (CS-ATP14, Fig. 1, Table 1). When the ATP dose increased further, the improvement in dewatering performance was quite limited (Supporting Information Fig. S6) but the resultant sludge mass was accordingly constantly increased. In addition, ATP dosed after St-CTA could also obviously alleviate the restabilization effect^6,23,24^. When St-CTA was overdosed at the dose of 26.67 mg/gTSS, the FCMC improved from 88.88 to 76.71% at the ATP dose of 14.00 kg/m^3^ (CS-ATP19, Fig. 1 and Table 1). The negatively surface charged solid particulates of ATP could act as a skeleton builder to reduce the compressibility of sludge and further improve the FCMC (Figs. 1a, 2), besides, could neutralize and combine the excessive positive charges of overdosed St-CTA and thus weaken the restabilization effect^6,23,24^ (CS-ATP15, CS-ATP17 and CS-ATP 19, Fig. 3). The formed composites of St-CTA and ATP may cause the flocs rough and compact (Supporting Information Fig. S7) due to the dense structure of ATP^25^. However, ATP had less improvement in some dewatering performance especially for CST after combination of St-CTA (Fig. 1c) possibly due to the error in the determination of the filtration volume and CST^26^ caused by decreased filtration time and the sludge cake broken quickly in the promoted dewatering processes after the addition of ATP.

In addition, the dewatering performance of this combination has been compared with a commercial flocculant, PAM, currently used in the wastewater treatment plant in Nanjing (Supporting Information Fig. S8). According to Fig. S8, the optimal FCMC obtained by PAM was 84.81% and was much higher than that by the combined conditioning method in this work approximately 76.99% (CS-ATP14, Fig. 1 and Table 1), although PAM had a lower optimal dose of 5.33 mg/gTSS. Fig. S8 still indicates that St-CTA in conjunction with ATP showed a wider effective dewatering window approximately 5.00–26.00 mg/g TSS than PAM about 3.00–8.00 mg/g TSS. Table 2 further compared their cost. Accordingly, the cost of the optimal conditioning process (CS-ATP14) was only 14.55 USD/tTSS, which was lower than PAM about 21.32 USD/tTSS in water plants. In addition to PAM, the dewatering performance of this combination has been roughly compared with some traditional coagulants also, such as FeCl_3_, polyaluminium chloride (PAC), and CaO, according to previous reports^19,27,28^. The optimal FCMC of sludge conditioned by FeCl_3_ or PAC alone was about 84%^19,27^, which was notably higher that by this combination. CaO had a good dewatering effect, but the resultant pH of the conditioned sludge was higher and the subsequent treatment was thus complicated^28^.

**Table 2:** Cost estimation of various flocculants/skeleton builder and their combinations including the cost of synthesis process.

Constituent	Unit price (USD/t)	Optimal dose (mg/g TSS)	Cost (USD/tTSS)
NaOH (Industrial grade)	500		
Corn starch	400		
CTA (Industrial grade)	1300		
St-CTA (Starch:CTA = 1:1.5)	879	16.0	14.24
ATP	33	9.3	0.31
St-CTA + ATP		16.0 + 9.3	14.55
PAM	4000	5.33	21.32

In short, St-CTA followed by the addition of ATP exhibited better and more stable dewatering performance because of the charge neutralization and bridging effects of St-CTA in conjunction of the skeleton builder effect of ATP, and the optimal doses of St-CTA and ATP were obtained about 16.00 mg/gTSS and 14.00 kg/m^3^, respectively.

### Sludge flocs and cakes

#### Sludge flocs

**Figure 4:**
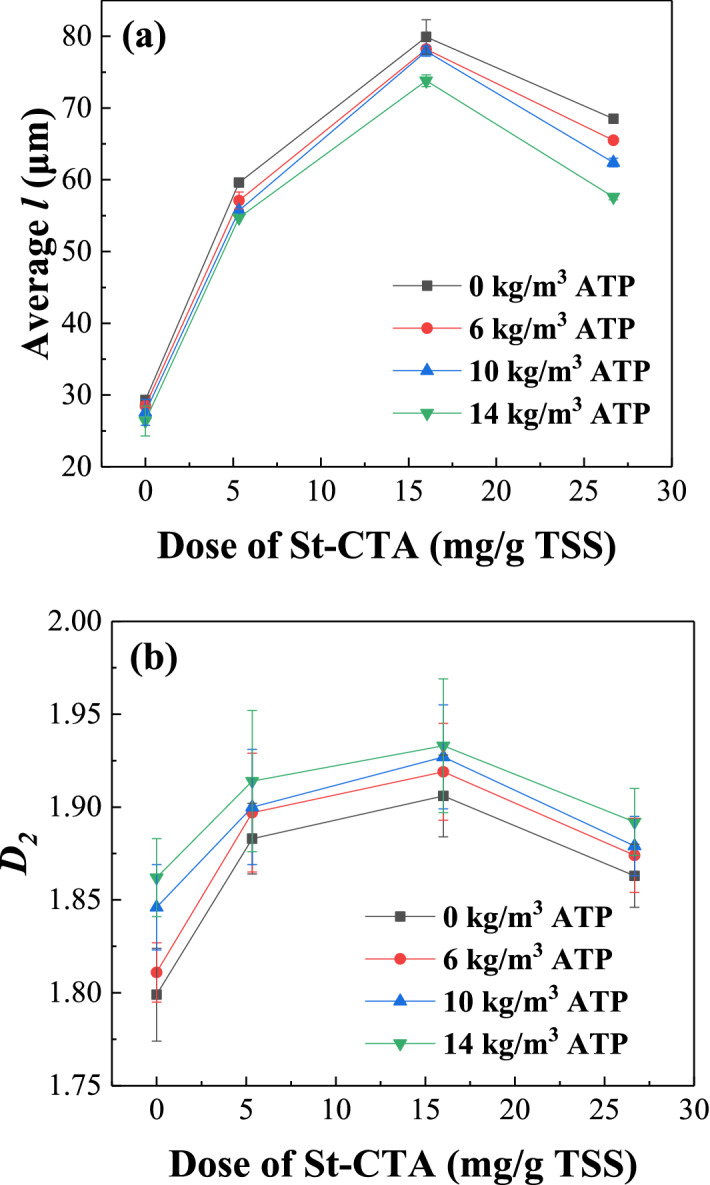
The properties of sludge flocs produced by St-CTA in conjunction with ATP using different doses: (**a**) Average floc size (*l*) and (**b**) 2D fractal dimension (*D*_2_).

**Figure 5:**
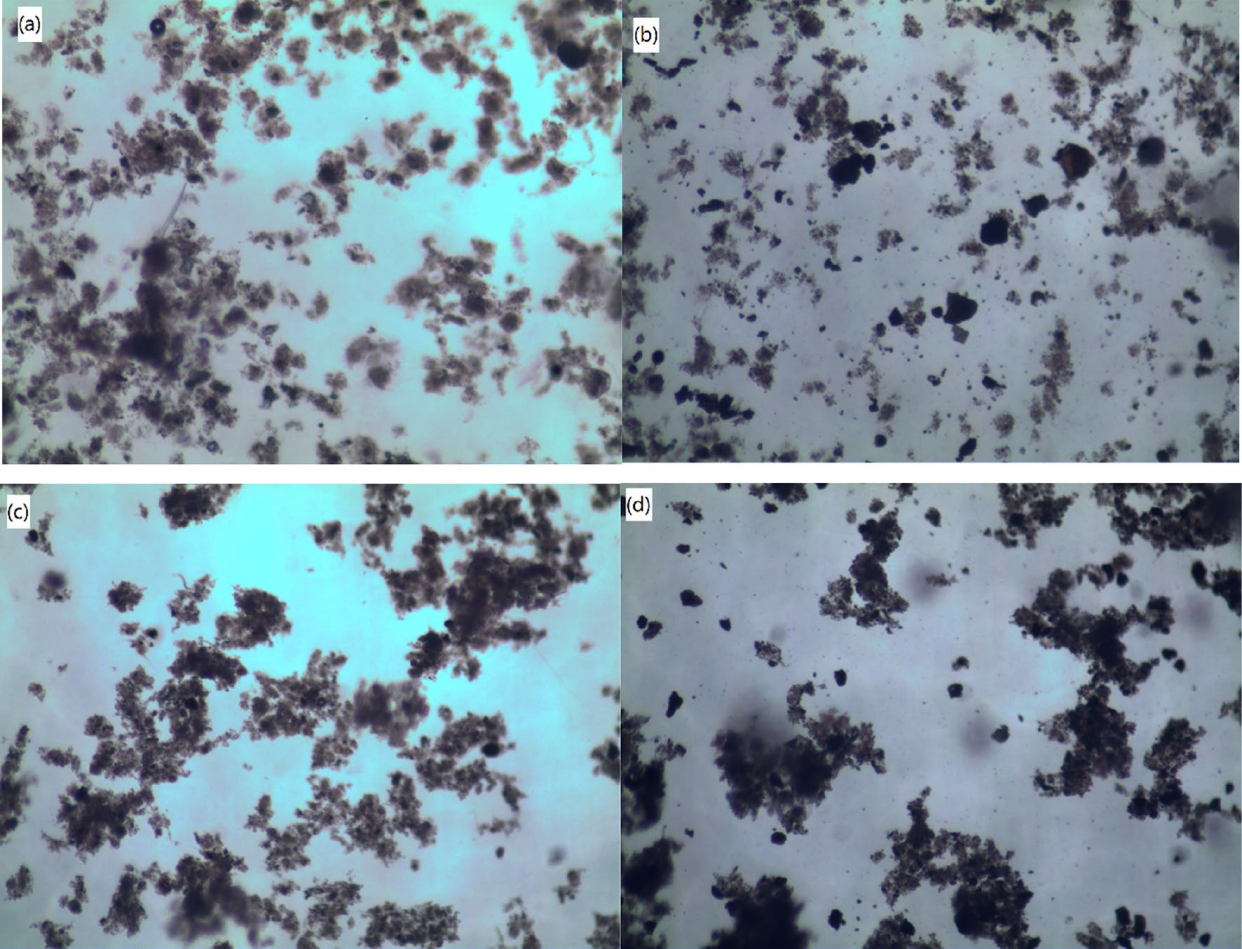
Optical microscope images of sludge flocs: (**a**) raw sludge and conditioned by (**b**) ATP, (**c**) St-CTA, and (**d**) St-CTA in conjunction with ATP at respective optimal dose, that is, St-CTA is 16.00 mg/gTSS and ATP is 14.00 kg/m^3^.

**Figure 6:**
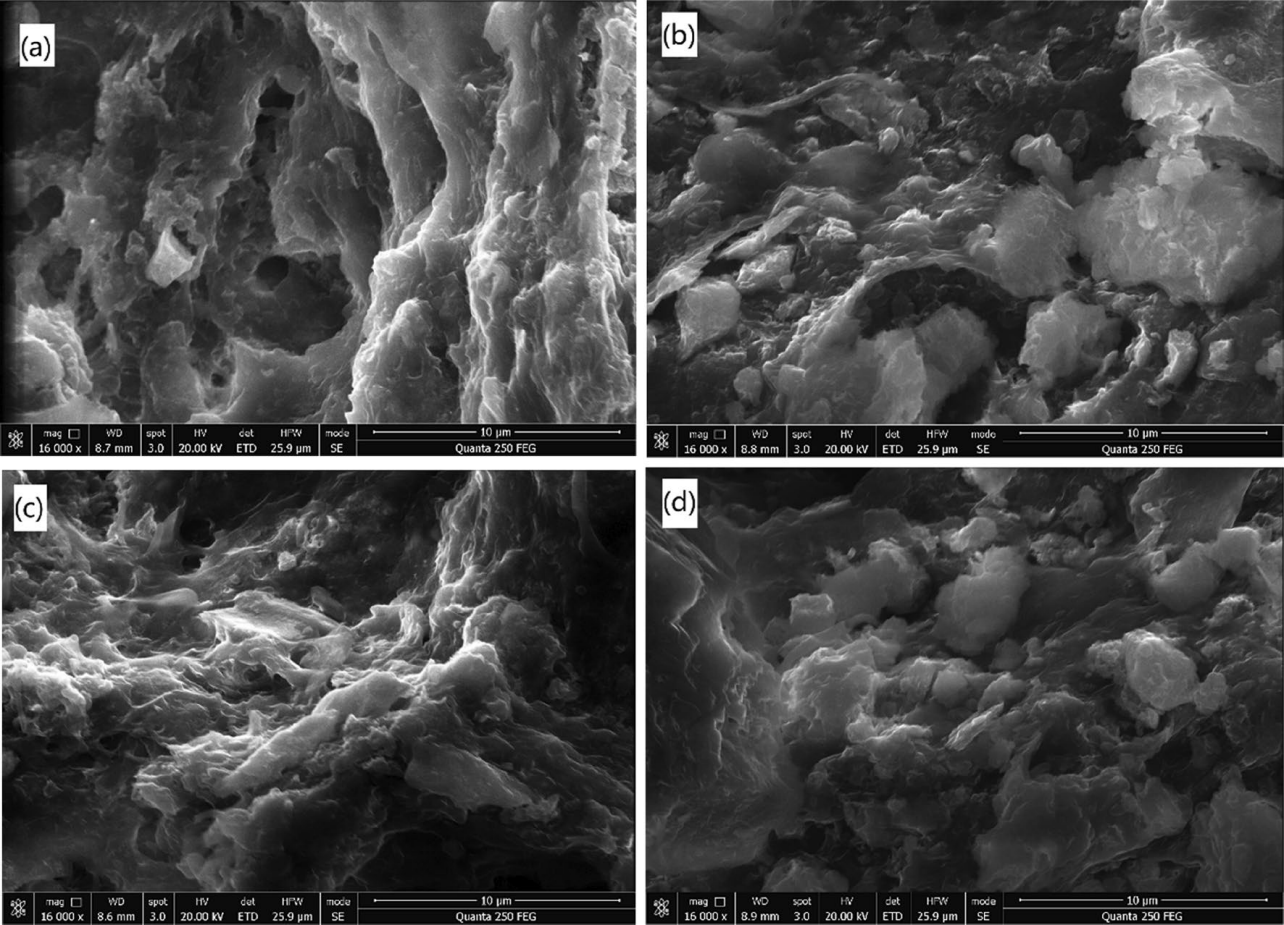
SEM images of sludge flocs: (**a**) raw sludge and conditioned by (**b**) ATP, (**c**) St-CTA, and (**d**) St-CTA in conjunction with ATP at respective optimal dose, that is, St-CTA is 16.00 mg/gTSS and ATP is 14.00 kg/m^3^.

The sizes and *D*_2_s of sludge flocs conditioned with different doses of St-CTA and ATP are shown in Fig. 4 based on the image analysis^7,29^ (Fig. 5 and Supporting Information Fig. S9). Figure 4 shows that the sizes and *D*_2_s of sludge flocs increased with the increase of the dose of St-CTA at the beginning but then decreased after reaching the maximal values at 16.00 mg St-CTA/g TSS, even in conjunction with ATP, due to the restabilization effect^6,23,24^, which was fully consistence with the dewatering performance (Figs. 1,2, Table 1, and Supporting Information Figs. S3–S5). In addition, the addition of St-CTA made the surface of sludge flocs turn from relatively smooth to rough and porous due to the efficient coagulation effect of St-CTA (Fig. 6c), which facilitated the aggregation and compression in the following mechanical dewatering to further remove the water from the sludge. Furthermore, the addition of ATP caused much more small solid particles embedded in the sludge flocs (Fig. 6b,d), as skeleton builders, to possibly create drainage channels in the subsequently formed sludge cakes and further improve the sludge dewatering performance^30^.

However, the sizes and *D*_2_s of sludge flocs had a contrary change trend with the increase of ATP doses after combined with St-CTA (Fig. 4). The addition of ATP made floc size smaller was possibly due to two facts. One was that the primary sludge flocs would be broken up by the second rapid mixing in the conditioning of ATP after St-CTA; The other was that some of ATP was not combined with the sludge flocs, and the employed ATP has an average particle size of approximately 18.107 μm, much smaller than the primary sludge flocs, which caused the obtained apparent average sludge flocs size turned smaller (Fig. 4). However, the denser structure of ATP was resultant in more compact internal structures of sludge flocs and higher detected *D*_2_s^31^. Consistently, the addition of ATP after St-CTA could benefit to the improvement of the dewatering performance (Figs. 1,2, Table 1, and Supporting Information Figs. S3–S5), which implicated that the relatively small but compact sludge flocs caused a good dewatering property (Fig. 4).

#### Sludge cakes

**Figure 7:**
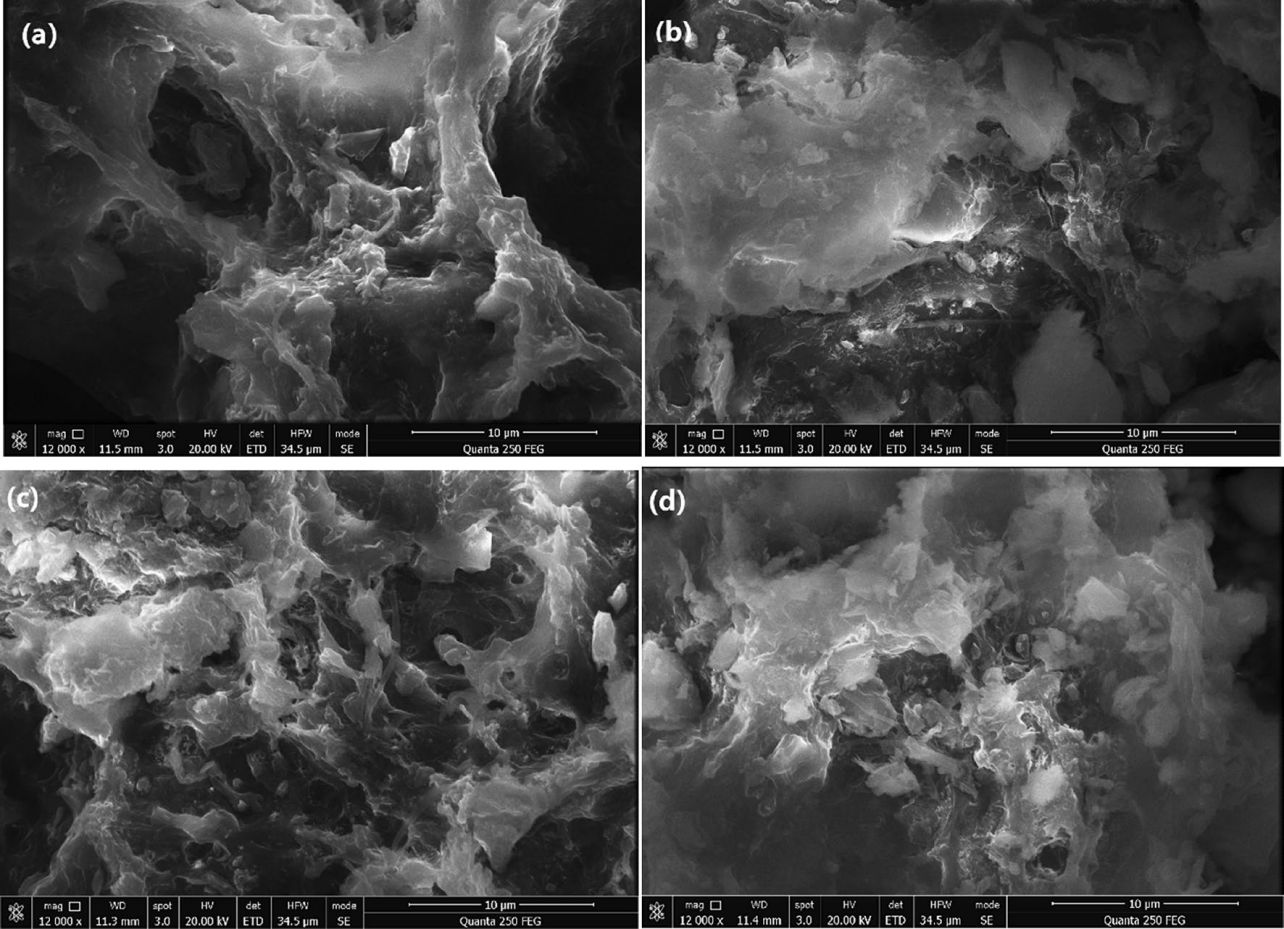
SEM images of sludge cakes: (**a**) raw sludge and conditioned by (**b**) ATP, (**c**) St-CTA, and (**d**) St-CTA in conjunction with ATP at respective optimal dose, that is, St-CTA is 16.00 mg/gTSS and ATP is 14.00 kg/m^3^.

The sludge flocs were further aggregated, compressed and formed into sludge cakes under the following mechanical squeezing. Figure 7 compares the surface morphologies of sludge cakes obtained without and with conditioning under various treatments. The surface of the sludge cake without conditioning was relatively smooth, flat and lacking micropores (Fig. 7a). After conditioning by St-CTA, ATP and their combination, respectively, the surfaces of the sludge cakes all became rough and microporous, among which this change in surface morphology of sludge cake treated by the combination of St-CTA and ATP was more evidently (Fig. 7b–d). This voids and porous structure could create drainage channels in sludge cakes and improve the filterability and permeability, which was beneficial to further discharge the internal water from the sludge^30,32^. The observed surface morphologies of sludge cakes were fully consistent with their corresponding sludge floc properties (Fig. 4), compression coefficients (Fig. 2 and Supporting Information Fig. S5 and dewatering performance (Fig. 1, Table 1, and Supporting Information Figs. S3–S4). St-CTA can efficiently coagulate and aggregate the sludge mainly by charge neutralization, and also partial St-CTA and ATP acted as skeleton builders can reduce the compressibility of the sludge cakes. Moreover, the superior dewatering performance of the combination of St-CTA and ATP was ascribed to their synergistic effects.

### EPS analysis

#### EPS fractions

**Figure 8:**
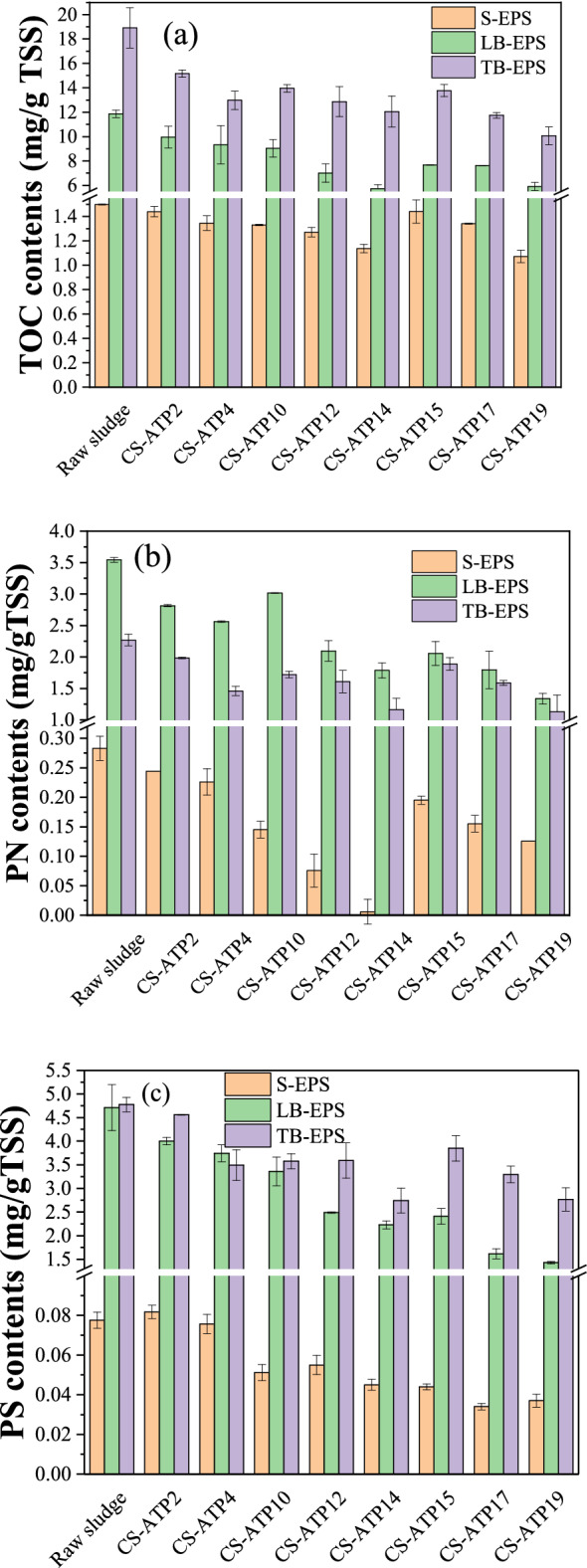
The contents of different EPS components in sludge conditioned by St-CTA in conjunction with ATP using different doses: (**a**) TOC, (**b**) protein (PN) and (**c**) polysaccharides (PS).

According to numerous reports in the literature^33,34^, EPS is one of the main factors affecting the sludge dewatering efficiency, but the effect of ATP on EPS has not been systematically studied. The effects of ATP on the EPS fractions and components were investigated (Fig. 8). As to the sludge conditioned by ATP only (CS-ATP2 and CS-ATP4) and St-CTA individually (CS-ATP10 and CS-ATP15), respectively, the TOC contents in three EPS factions (S-, LB- and TB-EPS) were almost declined (Fig. 8a). These findings indicated that St-CTA and ATP could both inhibit the EPS. The S-EPS would aggregate and settle down while part of LB-EPS would be converted into TB-EPS owing to the charge neutralization and bridging flocculation effects of St-CTA^19^ and the possible interactions of ATP, such as the chelation effect between the metal ions on ATP and those organic matters^35,36^, which would be discussed in detail in the following section. However, the further decreased contents of TB-EPS might be due to that they were bound to the sludge particles too tightly to detect under currently used measured methods^7,37–39^. Besides, the TOC contents in EPS were thus continuously decreased with the dose of ATP due to their enhanced interactions (CS-ATP2/CS-ATP4, CS-ATP10/CS-ATP12/CS-ATP14, and CS-ATP15/CS-ATP17/CS-ATP19). However, the TOC contents in three EPS factions had slightly increased due to the restabilization effect^19,23,24^ , when St-CTA was overdosed (CS-ATP15).

Moreover, St-CTA in conjunction with ATP caused the TOC contents in those three EPS factions decreased further because of their synergistic effect, confirming the superior dewatering effect of this combined technique (Fig. 8a). The optimal process, i.e. CS-ATP14, was accordingly obtained, in which the doses of ATP and St-CTA were 14.00 kg/m^3^ and 16.00 mg/g TSS, respectively. The changes in TOC contents in EPS with the doses of St-CTA and ATP were fully matched to the sludge dewatering performance (Figs. 1,2, Table 1, and Supporting Information Figs. S3–S5). The decrease in the EPS contents was confirmed to be beneficial to improvement of the dewaterability of sludge^33,34^.

### Components in different EPS fractions

#### PN and PS contents

The chemical compositions of EPS mainly include PN, PS, humic acid, fulvic acid, and nucleic acid, in which PN and PS are two important ones^40^. The contents of PN and PS in different EPS fractions of the sludge conditioned by different CS-ATP processes were further determined in Fig. 8b,c. Figure 8b,c show that the change trends in the contents of PN and PS were almost similar to those of TOC in three EPS fractions with the doses of St-CTA and ATP (Fig. 8a). Differently, the contents of PN in all three EPS fractions decreased evidently but those of PS was changed insignificantly to one another after the addition of ATP in the both presence and absence of St-CTA (CS-ATP2/CS-ATP4, CS-ATP10/CS-ATP12/CS-ATP14, and CS-ATP15/CS-ATP17/CS-ATP19). This finding indicated that ATP mainly acted on the PN rather than PS, because the metal irons on ATP, such as Al^3+^ and Fe^3+^ which initially bind to the negatively surface charged ATP, would readily chelate with the –NH_2_ and –COOH of PN, resulting in the further aggregation and precipitation of PN^35,36^. Moreover, PN in S-EPS was substantially inhibited in the optimal process, i.e. CS-ATP14, confirming the efficient synergistic effects of St-CTA and ATP.

Besides, the strong Pearson correlations between PN, PS and TOC contents of different EPS fractions and the FCMC and SRF of sludge as shown in Table 3 also indicated the PN, PS and TOC contents in EPS were closely related to the sludge dewatering performance^41^. In short, St-CTA in conjunction with ATP could efficiently inhibit the EPS and thus effectively improve the dewaterability of sludge.

**Table 3:** Pearson correlation between the sludge dewaterability and the PN, PS and TOC contents or the intensities of various 3D fluorescent signals in different EPS fractions.

EPS fractions			SRF			FCMC		
			*R*^2^	*p*	*n*	*R*^2^	*p*	*n*
S-EPS	TOC		0.687*	0.041	9	0..839**	0.005	9
	PS		0.913**	0.001	9	0.877	0.002	9
	PN		0.851**	0.004	9	0.896**	0.001	9
	λex/em	230/340	0.916**	0.001	9	0.900**	0.001	9
		280/350	0.932**	0.000	9	0.911**	0.001	9
		240/420	0.856**	0.003	9	0.872**	0.002	9
		350/440	0.880**	0.002	9	0.910**	0.001	9
		270/450	0.897**	0.001	9	0.937**	0.000	9
LB-EPS	TOC		0.863**	0.003	9	0.891**	0.001	9
	PS		0.870**	0.002	9	0.875**	0.002	9
	PN		0.736*	0.024	9	0.775*	0.014	9
	λex/em	230/340	0.879**	0.002	9	0.874**	0.002	9
		280/350	0.928**	0.000	9	0.937**	0.000	9
		240/420	0.669*	0.049	9	0.617	0.077	9
		350/440	0.052	0.894	9	0.271	0.480	9
		270/450	0.518	0.154	9	0.458	0.215	9
TB-EPS	TOC		0.761*	0.017	9	0.800**	0.010	9
	PS		0.800**	0.010	9	0.873**	0.002	9
	PN		0.657*	0.050	9	0.776*	0.014	9
	λex/em	230/340	0.885**	0.002	9	0.942**	0.000	9
		280/350	0.903**	0.001	9	0.856**	0.003	9
		240/420	0.730*	0.026	9	0.778*	0.014	9
		350/440	0.630	0.069	9	0.724*	0.028	9
		270/450	0.560	0.117	9	0.649	0.059	9

*Correlation is significant at the 0.05 level (2-tailed).

**Correlation is significant at the 0.01 level (2-tailed).

### 3D-EEM analysis

As mentioned earlier, the EPS still contains many other organic substances in addition to PN and PS. The 3D-EEM spectra of different EPS fractions in sludge before and after conditioned by St-CTA and ATP with different doses were also measured (Supporting Information Figs. S10, S11). According to previous literature^42,43^, the 3D-EEM spectrum can be mainly divided into five regions representing different substances, mainly aromatic PN (λ_ex/em_ = 230/340 nm, Peak A), tryptophan-like PN (λ_ex/em_ = 280/350 nm, Peak B), fulvic acid (λ_ex/em_ = 240/420 nm, Peak C) and humic acid substances (λ_ex/em_ = 350/440 nm and 270/450 nm, Peaks D and E). Accordingly, Fig. 6 and Supporting Information Table S1 show the summary of the intensities of these five characteristic peaks in different EPS fractions.

**Figure 9:**
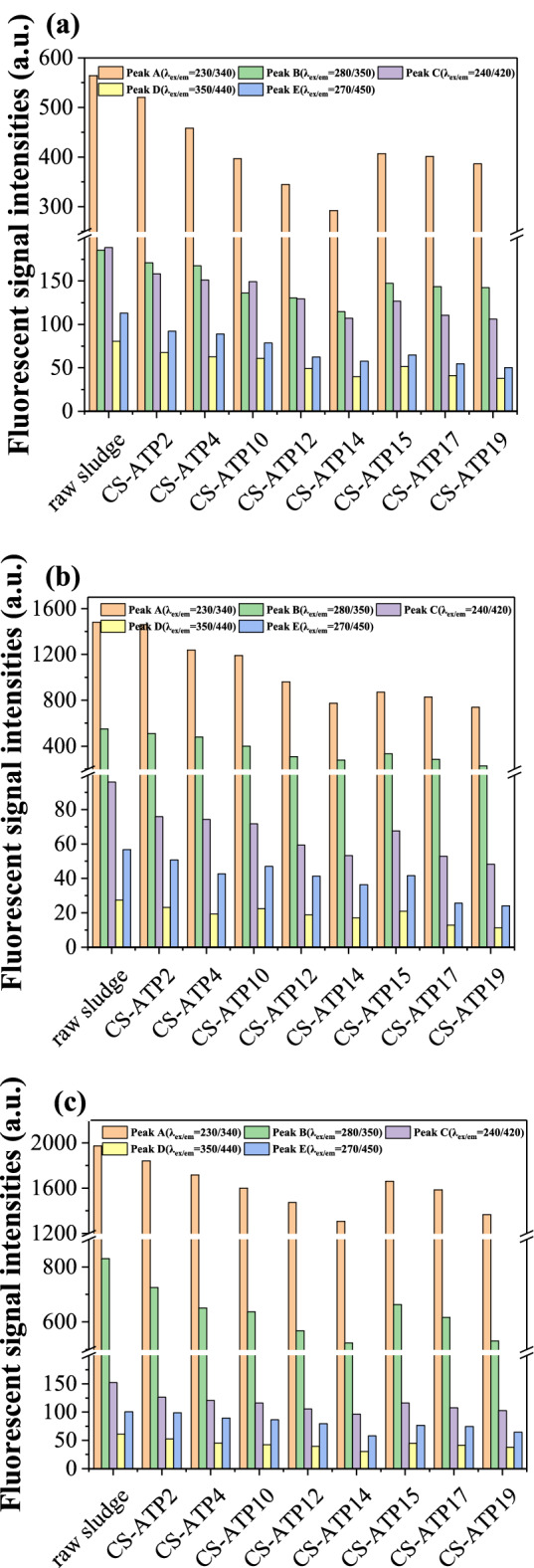
The intensities of various 3D-EEM characteristic peaks of (**a**) S-EPS, (**b**) LB-EPS and (**c**) TB-EPS in sludge conditioned by St-CTA in conjunction with ATP using different doses.

According to Fig. 9 and Supporting Information Table S1, the changes trends in the intensities of these five characteristic peaks in different EPS fractions with the doses of St-CTA and ATP were apparently similar to those in the TOC of the three EPS fractions (Fig. 8a). More detailly, the Pearson correlations between the intensities of the various fluorescent signals and the sludge dewaterability were shown in Table 3. Table 3 indicates that the protein-like substances including aromatic PN (Peak A) and tryptophan-like PN (Peak B) are both strongly related to the sludge dewatering performance in all three EPS fractions; however, the fulvic acid (Peak C) and humic acid substances (Peaks D and E) are closely associated with the sludge dewatering performance only in S-EPS. Combination of the correlation analysis of the TOC, PS and PN contents in Table 3, S-EPS is closely related to sludge dewatering performance^19^ and the PN in EPS rather than humic acid and fulvic acid has dominant effect^44–46^.

**Figure 10:**
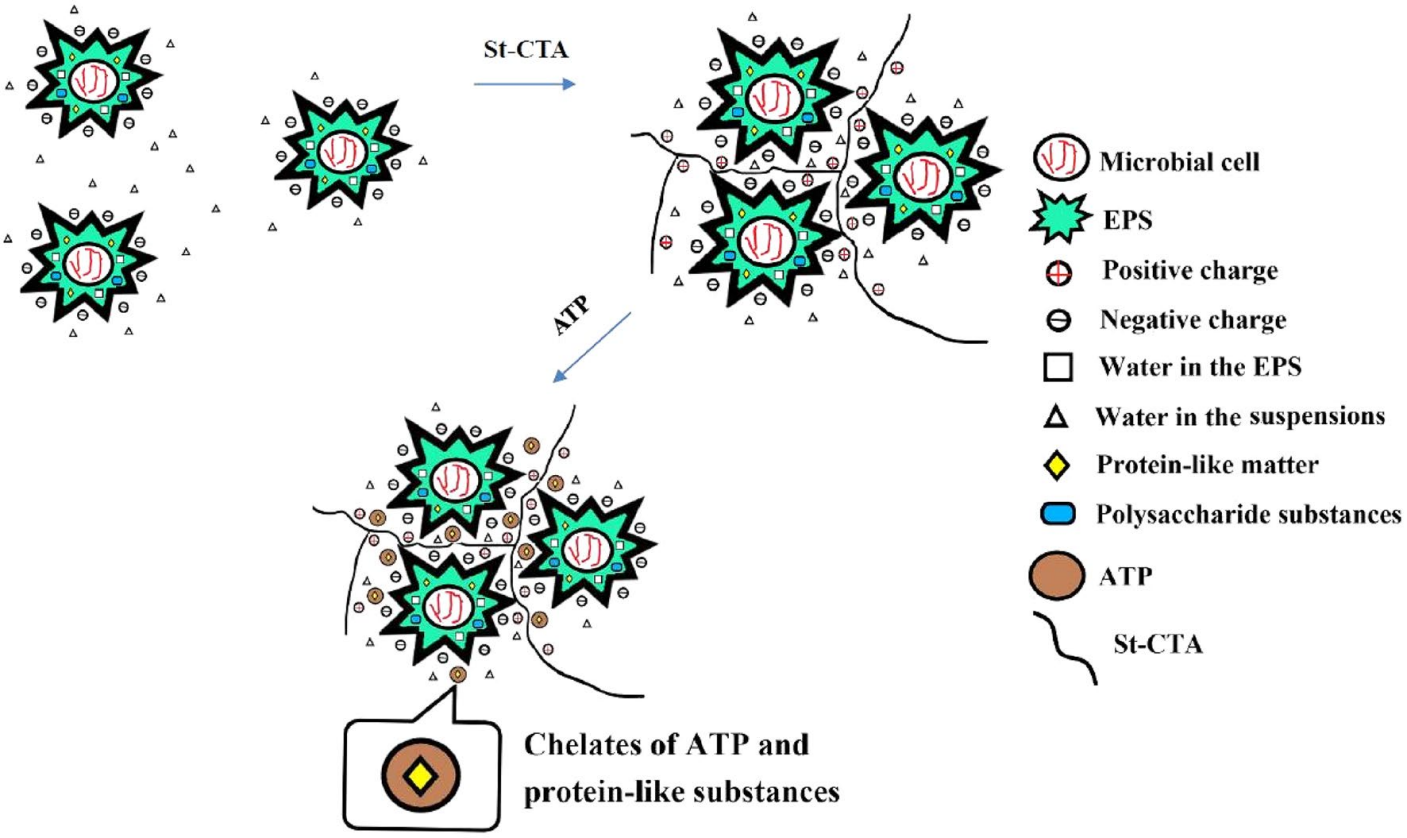
Schematic diagram of the mechanisms in sludge dewatering by the combination of St-CTA and ATP.

According to the aforementioned discussion, the sludge dewatering mechanisms were schematically described in Fig. 10. The negatively surface charged sludge particles combined with EPS were initially small and dispersed, in which contained a large amount of water. When the positively charged St-CTA was dosed, St-CTA would agglomerate the sludge particles and also efficiently inhibit the EPS together through charge neutralization and bridging flocculation effects. The following addition of ATP not only acted as a skeleton builder in the agglomerated sludge cakes to enhance the permeability and filterability of sludge but also effectively chelate with the highly hydrophilic PN substances in the EPS of the sludge through the metal ions on ATP, thereby together improvement of the dewaterability of the sludge^38,40,47^.

In addition to the aforementioned synergistic effects of St-CTA and ATP causing an efficient performance in sludge dewatering, the precursor of St-CTA, i.e. starch, and ATP are both natural materials with the evident features of environmental-friendliness, wide source and low cost. The combination of St-CTA and ATP thus had a high cost performance in sludge dewatering. However, there still exists some disadvantages and challenges in this combined conditioning method in the future application. The addition of ATP would inevitably increase the resultant total mass of sludge and thus increase the difficulty of the subsequent treatment. Besides, St-CTA and ATP could not substantially destroy the EPS in sludge and fully release the bound water, which caused to difficultly improve the sludge dewatering performance further. To reduce the dose of skeleton builder and the resultant sludge mass, high-performance flocculants should be developed, which are intrinsically based on the well-established structure–activity relationship^6,7^. Dewatering mechanisms should be thus studied in detail on the basis of the structural characteristics of flocculants and also the key components in sludge, such as the secondary structure of protein in EPS^48^. Besides, the pre-oxidation method may completely destroy the EPS in sludge^13^, and thus the combination process with pre-oxidation was feasible to further improve the sludge dewatering performance.

## Conclusions

This study mainly focused on the usage of a cationic etherified modified starch-based flocculant, St-CTA, followed by a clay material, ATP, to condition the sludge and improve the sludge dewatering; and the synergistic dewatering mechanisms were investigated in detail. The main results obtained were summarized as follows:

- (1) It demonstrated that ATP fed after St-CTA shows slightly higher efficiency in sludge dewatering than the other two dosing sequences. Specifically, the sludge conditioning process by 16.00 mg/g TSS of St-CTA in conjunction with 14.00 kg/m^3^ of ATP has a superior sludge dewatering performance and a low cost of approximately 14.55 USD/tTSS, besides, the FCMC was accordingly reduced from 97.86 to 76.99% and the sludge flocs were compacted with a *D*_2_ of approximately 1.933. The utilization of this combined conditioning process for sludge dewatering thus has a promising application potential.
- (2) On the basis of the analysis of the changes in the contents and distributions of the EPS fractions and components in the sludge conditioned by St-CTA and ATP with different doses associated with the Pearson correlation analysis, St-CTA and ATP could both inhibit the EPS, and S-EPS is closely related to sludge dewatering performance. Besides, the combination of St-CTA and ATP efficiently reduced the PN in all three EPS fractions totally from 6.09 to 3.01 mg/gTSS, which all exhibited significant correlations (*p* < 0.05) with the dewatering parameters of SRF and FCMC, and PN in EPS rather than humic acid and fulvic acid thus has a dominant effect in sludge dewatering.
- (3) The superior sludge dewatering performance of this combined process by St-CTA and ATP was ascribed to their synergistic effects. The positively charged St-CTA could efficiently aggregate and coagulate the sludge particles and also efficiently inhibit the EPS together through charge neutralization and bridging flocculation effects. The following addition of ATP not only acted as a skeleton builder in the agglomerated sludge cakes to enhance its permeability and filterability, causing the compression coefficient of the sludge cakes reduced from 1.26 to 0.79, but also effectively chelate with the highly hydrophilic PN substances in the EPS of the sludge through the metal ions on ATP, thereby together improvement of the dewaterability of the sludge.

## Experiment

### Materials

Starch (St, weight-average molecular weight ~ 1.5 × 10^5^ g/mol) was obtained from Binzhou Jinhui Corn Development Co., Ltd. 3-chloro-2-hydroxypropyltrimethylamm-onium chloride (CTA, 60wt% in water) was purchased from Aladdin Industrial Corporation. St-CTA with a St to CTA feeding mass ratio of 1:1.5 was synthesized, of which charge density was determined approximately 1.875 mmol/g by colloidal titration^29,49^. ATP was purchased from MESB (Meishibo), Changzhou, with an average particle size [d(0.5)] of approximately 18.107 μm obtained by a laser diffraction particle size analyzer (Mastersizer 2000, Malvern, UK). ATP was fully dried in an oven before use, the zeta potential of which was determined to be approximately -9.08 ± 0.09 mV. PAM (weight-average molecular weight of approximately 1.0 × 10^7^ g/mol and CD of 1.06 ± 0.13 mmol/g), was obtained from Dongying Nuoer Chemical Co., Ltd.

Waste-activated sludge samples were taken from a wastewater treatment plant at Nanjing, which treats sludge by the activated sludge process and membrane bioreactors. The samples were stored in a refrigerator at 4 °C and the same series of experiments by using the same sludge were completed within 7 days. All the physicochemical properties of the sludge including their detailed determination methods are shown in Supporting Information Table S2.

### Sludge conditioning

The 250-mL jars were performed for the conditioning of 100 mL sludge suspension through a six-place programmed paddle mixer model of TA6 (Wuhan Hengling Tech. Co. Ltd.) at room temperature. The detailed conditioning process was as follows. Various volumes of the freshly prepared St-CTA solution (4.0 g/L) was added to the sludge suspensions; the mixture was stirred quickly at 250 rpm for 1.0 min, followed by a slow stirring at 50 rpm for 2.0 min; and then different amounts of ATP were added into the sludge mixtures, which were stirred quickly at 250 rpm for 30 s and then a slow stirring at 50 rpm for 3.5 min. The conditioned sludge was used for subsequent experiments to determine the FCMC, SRF, capillary suction time (CST), compression coefficient, floc properties including floc size and compactness, zeta potentials, and the fractions and components of EPS extractions. The aforementioned characterization methods are described in detail in Supporting Information Table S2. Each experiment was measured in triplicate, and the final results represented the values average with the relative error less than 5%.

### Measurements of dewatering performance

FCMC and SRF are two important parameters for evaluating the sludge dewatering performance, where SRF is the determination of sludge specific resistance by pumping the conditioned sludge through a 0.05 MPa pressure Brinell funnel. The extracted filtrate was collected in a 100 mL measuring cylinder, and the cylinder reading was recorded every 5 s from the start of pumping until the sludge cake broke or until it reached 6.0 min. The sludge cake was dried in an oven at 105 °C and the FCMC was determined according to previous report^50^. The SRF of the sludge was calculated as follows^7,49^:

1 $$SRF=\frac{2P{{S}_{a}}^{2}b}{\mu \omega },$$

where P (N/m^2^) represents the pressure used for filtration, *S*_*a*_ (m^2^) shows the area of the filter paper used for extraction, *b* (s/m^6^) indicates the slope of filtrate discharge curve; μ is the kinetic viscosity and ω indicates the dry weight per unit volume of sludge on the filtrate medium.

CST was measured by using a CST apparatus (England Triton Electronics 304 m) to indicate the filterability of free water in sludge^51^. The sludge compression performance is expressed by the sludge compression coefficient (*s*), which is obtained by measuring the sludge specific resistances under different pumping pressures, i.e. 0.02, 0.03, 0.04 and 0.05 MPa according to Eq. (2)^47,52^:

2 $$\frac{{SRF}_{1}}{{SRF}_{2}}={\left(\frac{{P}_{1}}{{P}_{2}}\right)}^{s}.$$

TTF refers to the time required to obtain a filtrate volume equal to the half volume of sludge under the pressure of 0.05 MPa^5,53^. The filtration volume refers to the volume of filtrate in the measuring cylinder at the end of the filtration. The filtrate rate (*q*), which indicates the rate of filtration, is determined by Darcy’s law according to Eq. (3):

3 $$q=\frac{V}{{S}_{b}\times t},$$

where *V* is a filtrate volume (m^3^) which is read from the measuring cylinder at the end of filtration, *S*_*b*_ is a filtration area (m^2^) and *t* is filtration time (h)^53^.

### Floc properties

The floc properties of the treated sludge including the floc size (*l*) and the two-dimensional fractal dimension (*D*_2_) were determined to study the microstructural changes in the conditioned sludge. The sludge flocs were photographed with a Pentax Model K-m digital camera equipped with an optical microscope (XTL-3400; Shanghai Caikon Optical Instrument Co., Ltd.) under a fixed magnification. *l* was the characteristic length of sludge floc and the longest line joining two points of object’s outline and passing through the centroid. As mentioned in the previous study^7,29^, the projected characteristic length *l* and the projected area (*A*) of the sludge floc were measured by means of an image analysis software (Image pro® Plus 6.0), and *D*_2_ was accordingly obtained by the logarithmic fitting of Eq. (4).

4 $$A\propto {l}^{{D}_{2}}.$$

The conditioned sludge flocs and their sludge cakes formed after a following mechanical squeezing were freeze-dried at − 60 °C for 72 h and then their surface morphologies were directly observed using a scanning electron microscopy (SEM, FEI Quanta 250).

### EPS characterization

#### EPS extraction

The EPS in sludge was mainly classified into soluble EPS (S-EPS), loosely bound EPS (LB-EPS) and tightly bound EPS (TB-EPS)^6^, which are extracted by a modified ultrasonic thermal extraction method in this work^7,54^. Details of operation method are as follows: 10 mL of the sludge was centrifuged in a tube at 3000 rpm for 10.0 min, the supernatant was extracted and filtered through a 0.45 μm filter membrane to obtain S-EPS. The remaining sludge was resuspended to 10 mL with 0.05% mass fraction NaCl solution, sonicated at 20 kHz for 2.0 min and then shaken in a shaker at 150 rpm for 10.0 min, followed by centrifugation at 5000 rpm for 10.0 min, the supernatant was extracted and filtered through a 0.45 μm membrane to obtain LB-EPS. The remaining sludge was resuspended in 0.05% mass fraction NaCl solution to 10 mL, sonicated at 20 kHz for 3.0 min, heated in a water bath at 60 °C for 30.0 min, and then centrifuged at 8000 rpm for 10.0 min to extract the supernatant and filtered through a 0.45 μm membrane to obtain TB-EPS.

#### EPS analysis

Total organic carbon (TOC) in EPS fractions was measured by a total organic carbon analyzer (Aurora 1030 W, USA) to indicate dissolved organic matters in the sludge. Protein (PN) contents were measured using a UV-2600A spectrometer (Unico USA) with bovine serum albumin (BSA) as the standard substance^55,56^. The three extracts of EPS and the standard solution of BSA were stained with the prepared solution of Coomassie Brilliant Blue G-250 and the absorbance of PN was measured by UV at 595 nm after standing for 2.0 min. Polysaccharides (PS) contents were analyzed using the anthrone method^57^. Because PS can react with anthrone to a blue-green solution. EPS and prepared glucose standard solutions can be heated and reacted with anthrone and the absorbance measured by UV at 620 nm.

Three-dimensional excitation emission matrix (3D-EEM) spectra were measured by a F-7000 fluorescence spectrophotometer (Hitachi, Japan). The specific experimental conditions are as follows: The wavelength of the emitted light ranges from 250 to 550 nm at 1 nm increment and the wavelength range of the excitation light is from 200 to 450 nm in an increment of 5 nm. The scanning speed is 2400 nm/min and the emission and excitation slit bandwidths are 5 nm^38^.

### Correlation analysis

Correlation analysis was carried out though Pearson correlation coefficient calculation module of IBM SPSS Statistics version 22.0 which was mainly used to quantify the correlation between FCMC or SRF values and different fractions in sludge EPS^58^.

## Supplementary Information

Supplementary Information.

## Data Availability

The datasets used and/or analysed during the current study available from the corresponding author on reasonable request.
