# Supplementary material for: The enhanced dewaterability of sludge by a starch-based flocculant combined with attapulgite
Source: Sci Rep. 2023 Jan 9;13:402. doi: 10.1038/s41598-023-27749-3 (PMC9829677; doi:10.1038/s41598-023-27749-3)
Supplement: Supplementary file 1 — Supplementary Information. [file 41598_2023_27749_MOESM1_ESM.pdf]

Supporting Information Cover Sheet

The enhanced dewaterability of sludge by a starch-based flocculant combined with attapulgite *

Shaohang Shen [a], Hua Wei [b], Yu Pan [a], Pan Hu [a], Hu Yang [a]**

[a.] State Key Laboratory of Pollution Control and Resource Reuse, School of the Environment, Nanjing University, Nanjing 210023, P. R. China

[b.] School of Ecology Technology and Engineering, Shanghai Institute of Technology, Shanghai 201418, P. R. China

Number of pages: 18

Number of tables: 2

Number of figures: 11

---

* Supported by the National Natural Science Foundation of China (grant no. 51978325).

** Corresponding author. Tel & Fax: 86-25-89681272, E-mail: yanghu@nju.edu.cn

Table of Contents

Index	Captions	Page
**Table S1**	Intensities of fluorescent signals of different EPS fractions after conditioning.	S4
**Table S2**	Characteristics of waste-activated sludge.	S5
**Figure S1**	Influence of different dosing sequence of St-CTA in conjunction with ATP on sludge dewatering performance, i.e., St-CTA dosed before, after, or together with ATP. The doses of ATP were kept constant at 2 kg/m^3, 6 kg/m^3 and 10 kg/m^3.	S6
**Figure S2**	Influences of grinding on (a-f) the sludge dewatering performance and (g) the particle size of ATP.	S7
**Figure S3**	The filtrate volumes of sludge conditioned by St-CTA in conjunction with ATP using different doses.	S8
**Figure S4**	The filtration rates (*q*) of sludge conditioned by St-CTA in conjunction with ATP using different doses.	S9
**Figure S5**	The compressibility coefficients of sludge conditioned by St-CTA in conjunction with ATP using different dose.	S10
**Figure S6**	The effect of ATP dose on the sludge dewatering performance at optimal dose of St-CTA.	S11
**Figure S7**	SEM images of (a) St-CTA, (b) ATP, and (c) St-CTA in conjunction with ATP.	S12
**Figure S8**	The sludge dewatering performance of St-CTA in conjunction with ATP by using different doses compared with PAM: (a) filter cake moisture content and (b) SRF.	S13
**Figure S9**	Image analysis of various sludge flocs conditioned by	S14- S15

	St-CTA in conjunction with ATP at various doses: (a) raw-sludge, (b) CS-ATP2, (c) CS-ATP3, (d)CS-ATP4, (e) CS-ATP5, (f) CS-ATP7, (g) CS-ATP8, (h) CS-ATP9, (i) CS-ATP10, (j) CS-ATP12, (k) CS-ATP13, (l) CS-ATP14, (m) CS-ATP15, (n) CS-ATP17, (o) CS-ATP18, and (p) CS-ATP19.	
**Figure S10**	3D-EEM spectra of raw sludge: (a) S-EPS, (b) LB-EPS, and (c) TB-EPS fractions, respectively.	S16
**Figure S11**	3D-EEM spectra of various EPS fractions in sludge conditioned by St-CTA in conjunction with ATP using different doses. (S-EPS samples were diluted by 10 times, while LB-EPS and TB-EPS samples were diluted by 50 times).	S17-S18

Table S1 Intensities of fluorescent signals of different EPS fractions after conditioning.

EPS	Samples	Dose of CS (mg/gTSS)	Dose of ATP (kg/m^3)	$\lambda_{ex/em}$				
				230/340	280/350	240/420	350/440	270/450
S-EPS	Raw sludge	0.00	0	564.18	185.41	188.59	80.520	113.04
	CS-ATP2	0.00	6	520.28	171.11	158.19	67.526	92.339
	CS-ATP4	0.00	14	458.08	167.61	151.19	62.483	88.976
	CS-ATP10	16.00	0	396.28	135.91	149.29	60.753	78.503
	CS-ATP12	16.00	6	344.51	130.64	129.42	49.063	62.376
	CS-ATP14	16.00	14	292.38	114.84	107.19	39.563	57.569
	CS-ATP15	26.67	0	406.58	147.31	126.79	51.336	64.649
	CS-ATP17	26.67	6	400.71	143.44	110.64	41.020	54.716
	CS-ATP19	26.67	14	386.18	142.31	106.14	37.998	50.346
LB-EPS	Raw sludge	0.00	0	1480.98	549.06	95.957	27.283	56.636
	CS-ATP2	0.00	6	1459.98	509.26	76.003	23.090	50.783
	CS-ATP4	0.00	14	1238.98	478.81	74.29	19.243	42.656
	CS-ATP10	16.00	0	1189.98	399.71	71.71	22.343	46.926
	CS-ATP12	16.00	6	960.98	307.78	59.36	18.700	41.286
	CS-ATP14	16.00	14	773.88	278.18	53.33	17.043	36.346
	CS-ATP15	26.67	0	870.28	333.18	67.587	20.820	41.591
	CS-ATP17	26.67	6	829.18	284.41	52.863	12.843	25.696
	CS-ATP19	26.67	14	739.28	228.41	48.217	11.210	24.129
TB-EPS	Raw sludge	0.00	0	1972.6	830.04	152.03	60.913	100.61
	CS-ATP2	0.00	6	1841.3	725.04	126.35	52.258	98.552
	CS-ATP4	0.00	14	1716.3	651.04	120.43	45.093	89.362
	CS-ATP10	16.00	0	1599.1	636.49	115.9	42.183	86.312
	CS-ATP12	16.00	6	1471.6	567.04	105.6	39.303	79.645
	CS-ATP14	16.00	14	1307.3	523.97	96.467	30.113	57.877
	CS-ATP15	26.67	0	1660.1	663.14	116.07	44.873	76.472
	CS-ATP17	26.67	6	1584.9	616.41	107.63	41.556	74.577
	CS-ATP19	26.67	14	1366.6	530.64	102.55	37.673	64.297

Table S2 Characteristics of waste-activated sludge.

Parameter	Value	Parameter	Value
Moisture content (%)	98.5	Cr (mg/L)	< 0.05
pH	6.8 ± 0.15	Cu (mg/L)	< 0.05
VSS/TSS	43.0 ± 0.9%	Fe (mg/L)	0.100
specific resistance of filtration (SRF) ($\times 10^{12}$ m/kg)	4.10±0.29	K (mg/L)	19.4
Zeta potential (mV)	-9.3±0.7	Mg (mg/L)	12.5
SCOD (mg/L)	44-78	Mn (mg/L)	0.163
Conductivity (mS/cm)	0.71 ± 0.09	Na (mg/L)	55.2
Al (mg/L)	0.033	Ni (mg/L)	< 0.05
Ba (mg/L)	< 0.05	Si (mg/L)	0.793
Ca (mg/L)	53.0	Sr (mg/L)	0.286
Cd (mg/L)	< 0.05	Zn (mg/L)	< 0.05
Co (mg/L)	< 0.05		

*The moisture content and the ratio of volatile suspended solids (VSS) to total suspended solids (TSS, VSS/TSS) were analyzed according to APHA (1998); the pH of the filtrate was measured using a Delta 320 pH meter (Mettler Toledo, Switzerland); the zeta potential and conductivity were detected by a Zetasizer Nano Z (Malvern, UK); COD was detected by $K_2Cr_2O_7$ titration method; Dissolved inorganic cations were using an optima 5300DV ICP-MS (PE, USA); FCMC and SRF was measured according to the **Experiment part**.

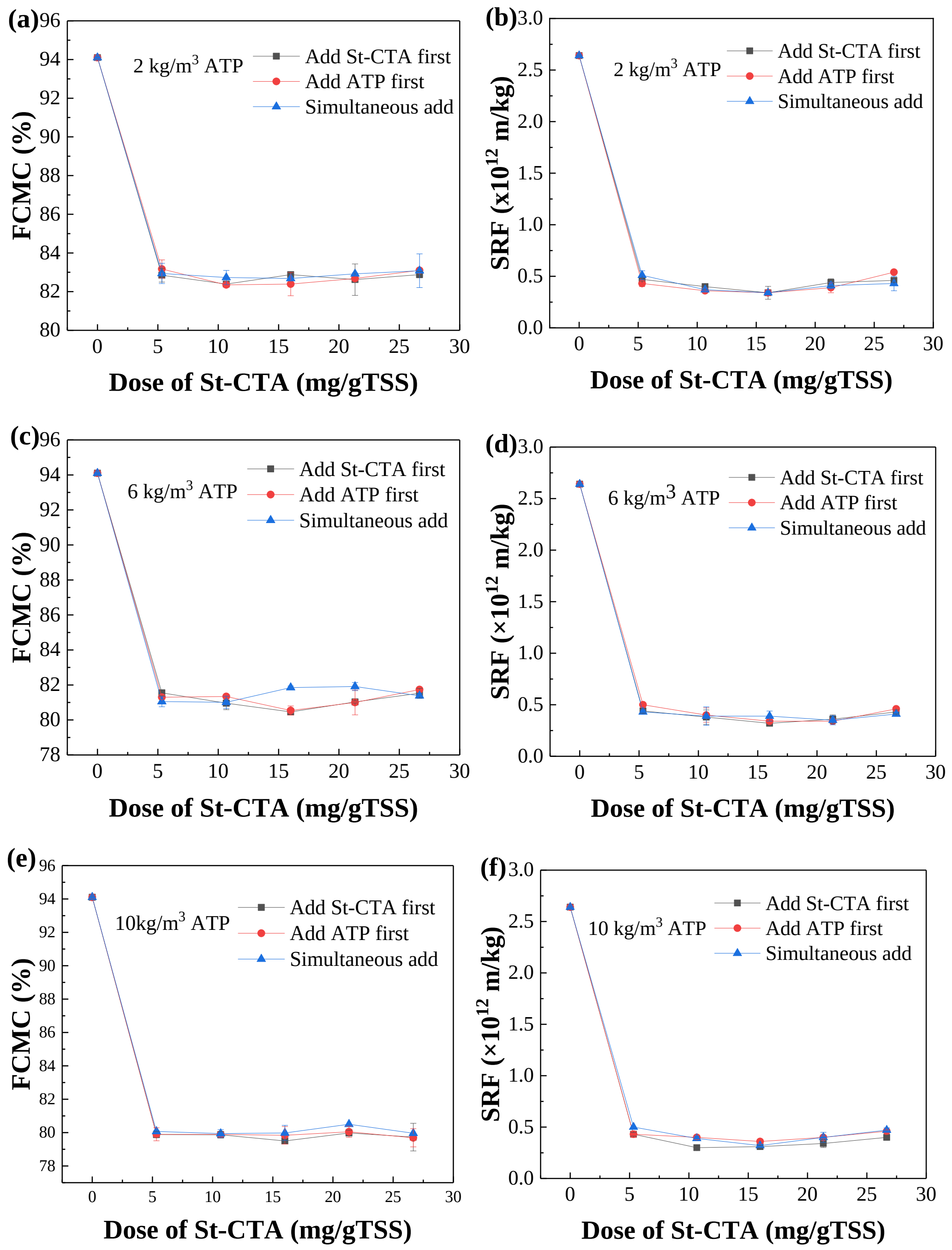

Figure S1 Influence of different dosing sequence of St-CTA in conjunction with ATP on sludge dewatering performance, i.e., St-CTA dosed before, after, or together with ATP. The doses of ATP were kept constant at 2 kg/m^3, 6 kg/m^3 and 10 kg/m^3.

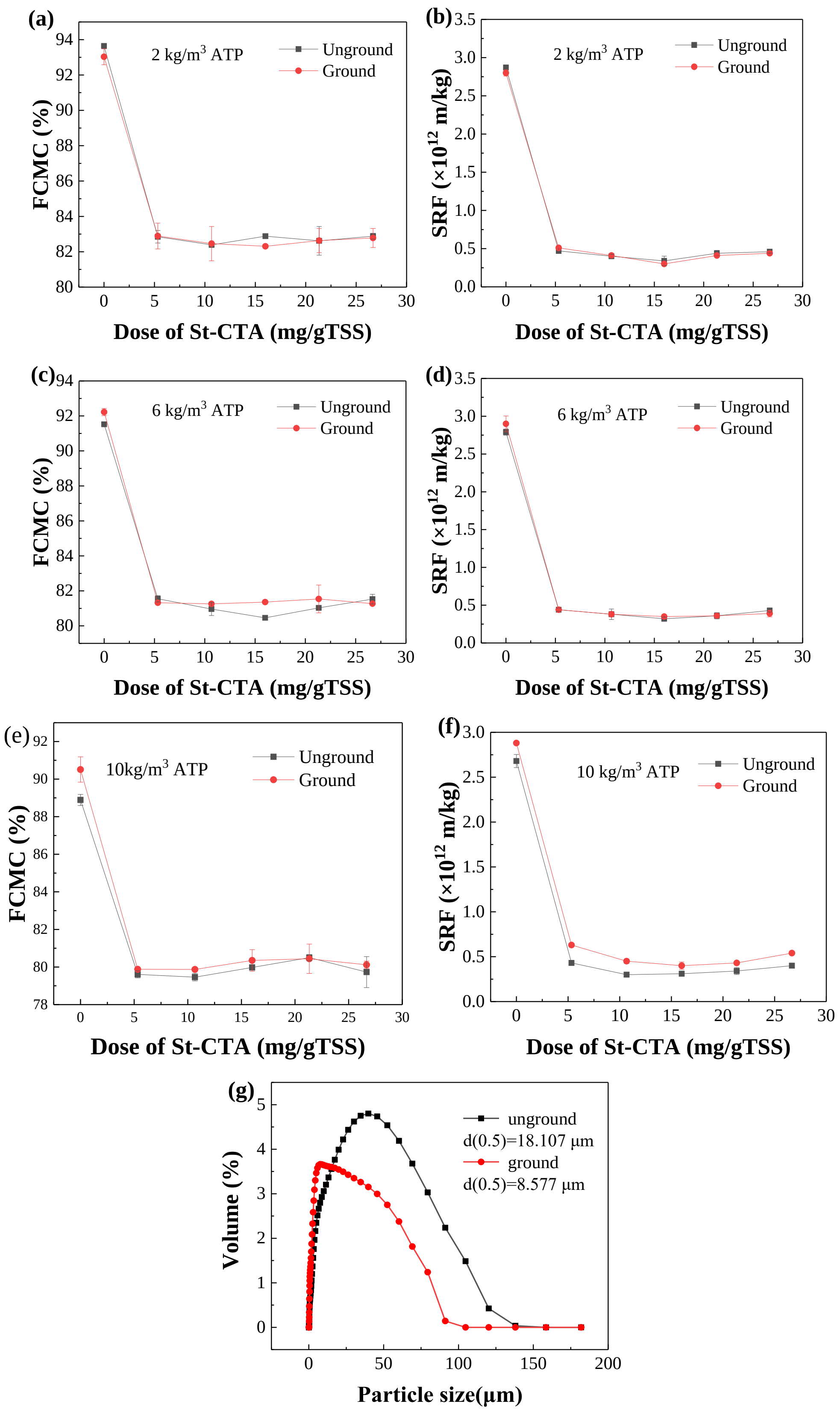

Figure S2 Influences of grinding on (a-f) the sludge dewatering performance and (g) the particle size of ATP.

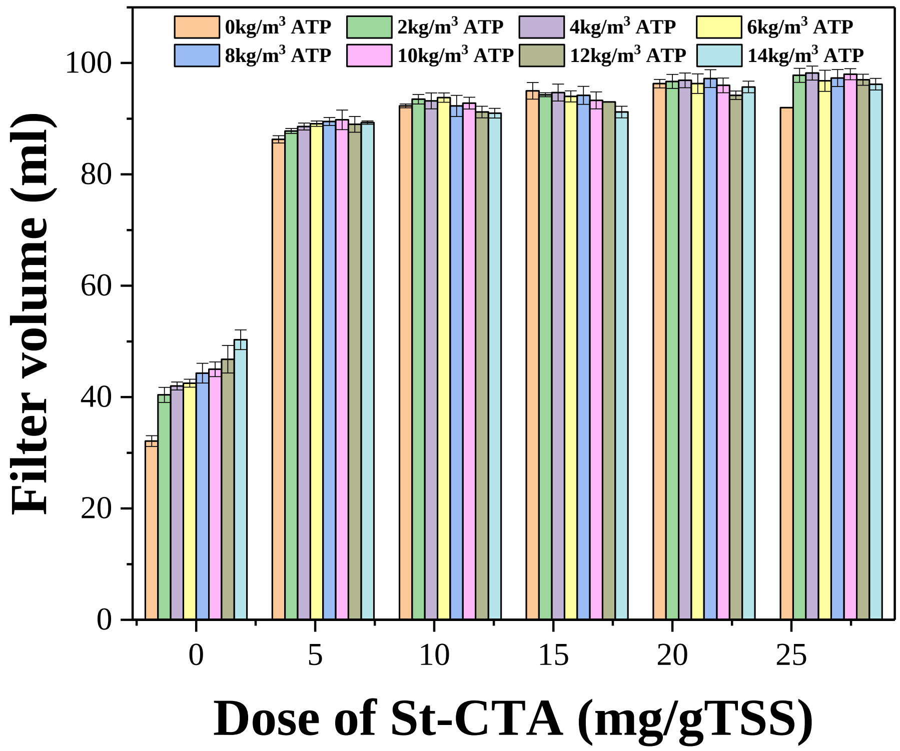

Figure S3 The filtrate volumes of sludge conditioned by St-CTA in conjunction with ATP using different doses.

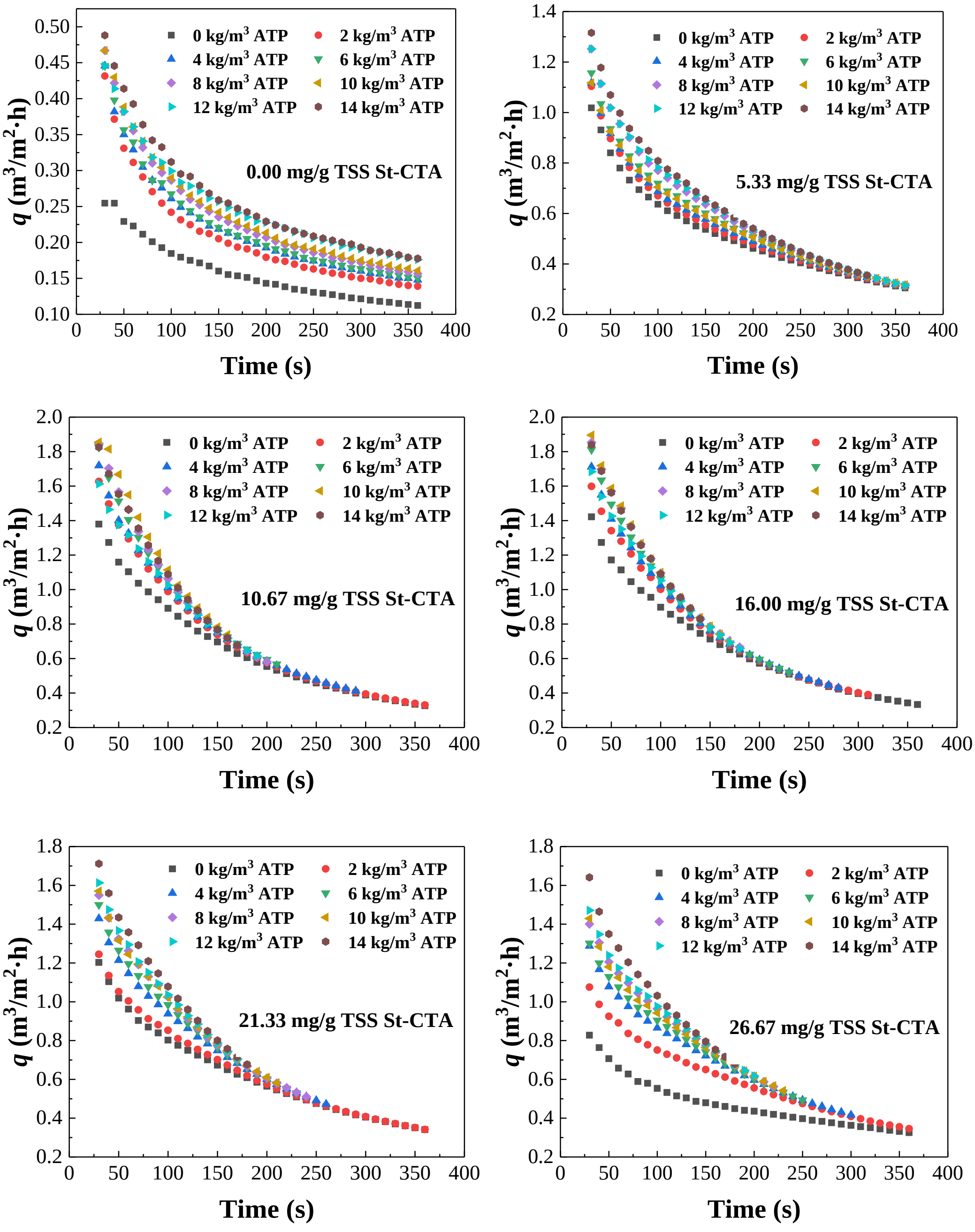

Figure S4 The filtration rates (q) of sludge conditioned by St-CTA in conjunction with ATP using different doses.

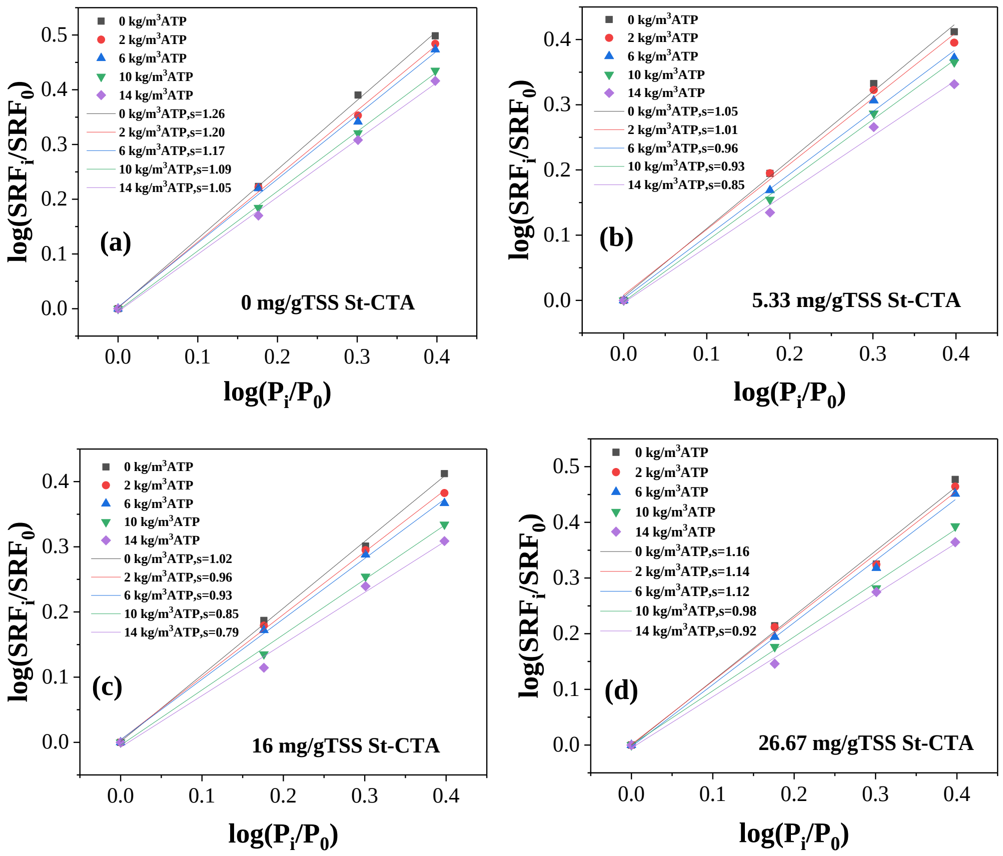

Figure S5 The compressibility coefficients of sludge conditioned by St-CTA in conjunction with ATP using different dose.

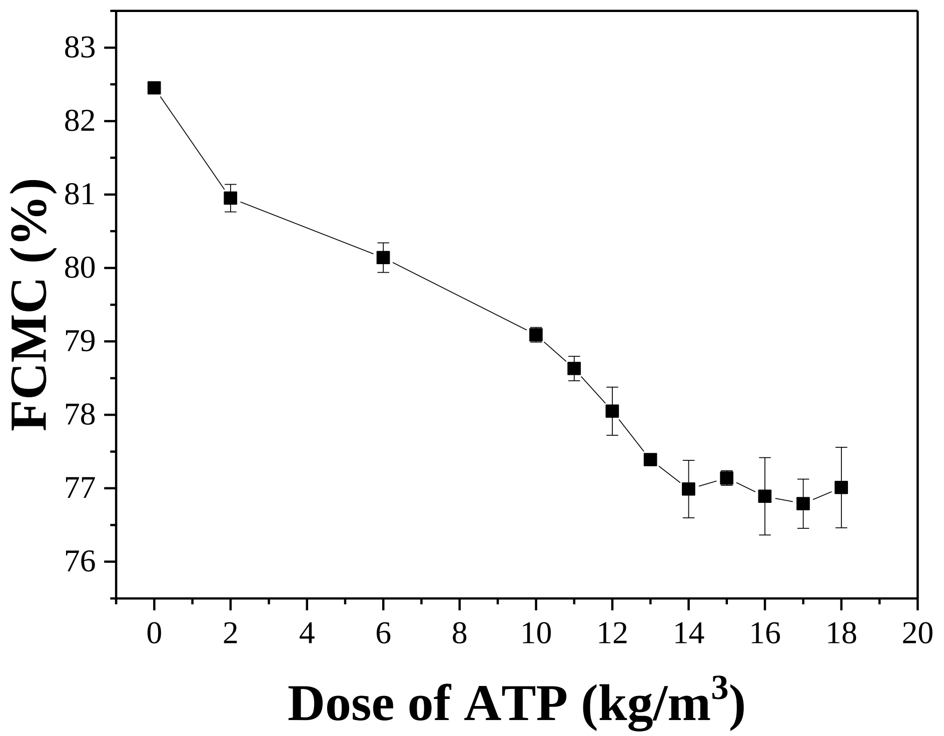

Figure S6 The effect of ATP dose on the sludge dewatering performance at optimal dose of St-CTA.

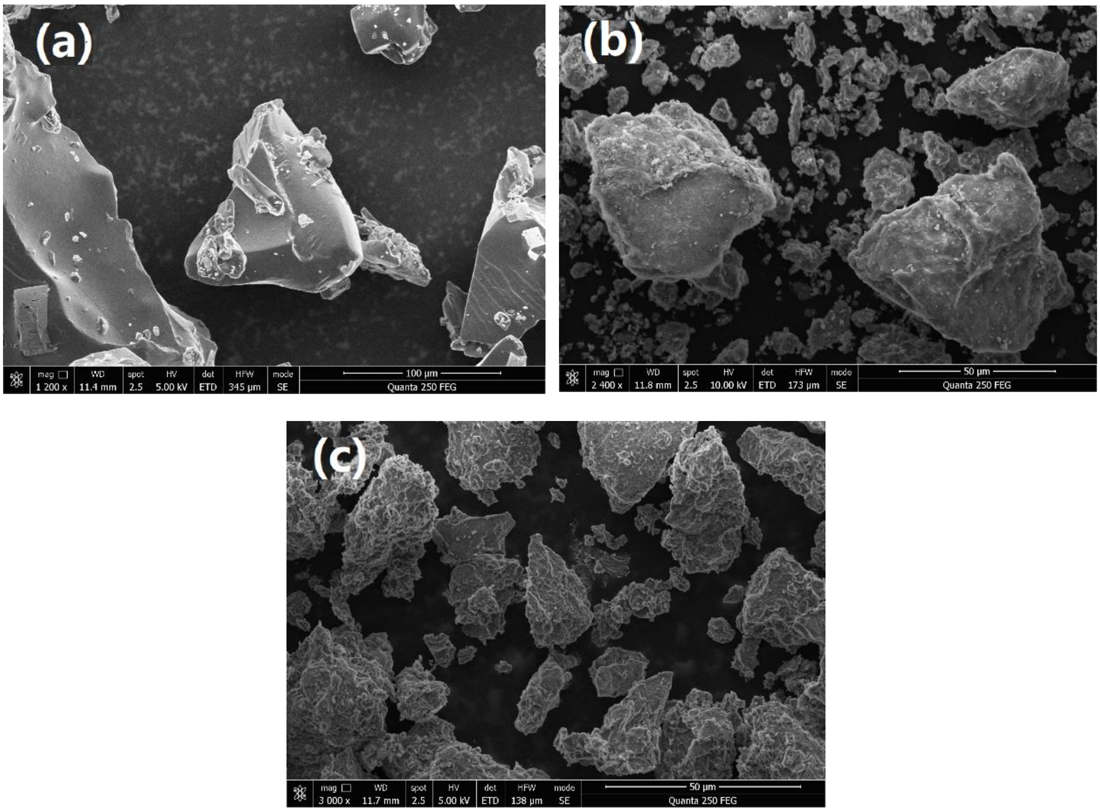

Figure S7 SEM images of (a) St-CTA, (b) ATP, and (c) St-CTA in conjunction with ATP.

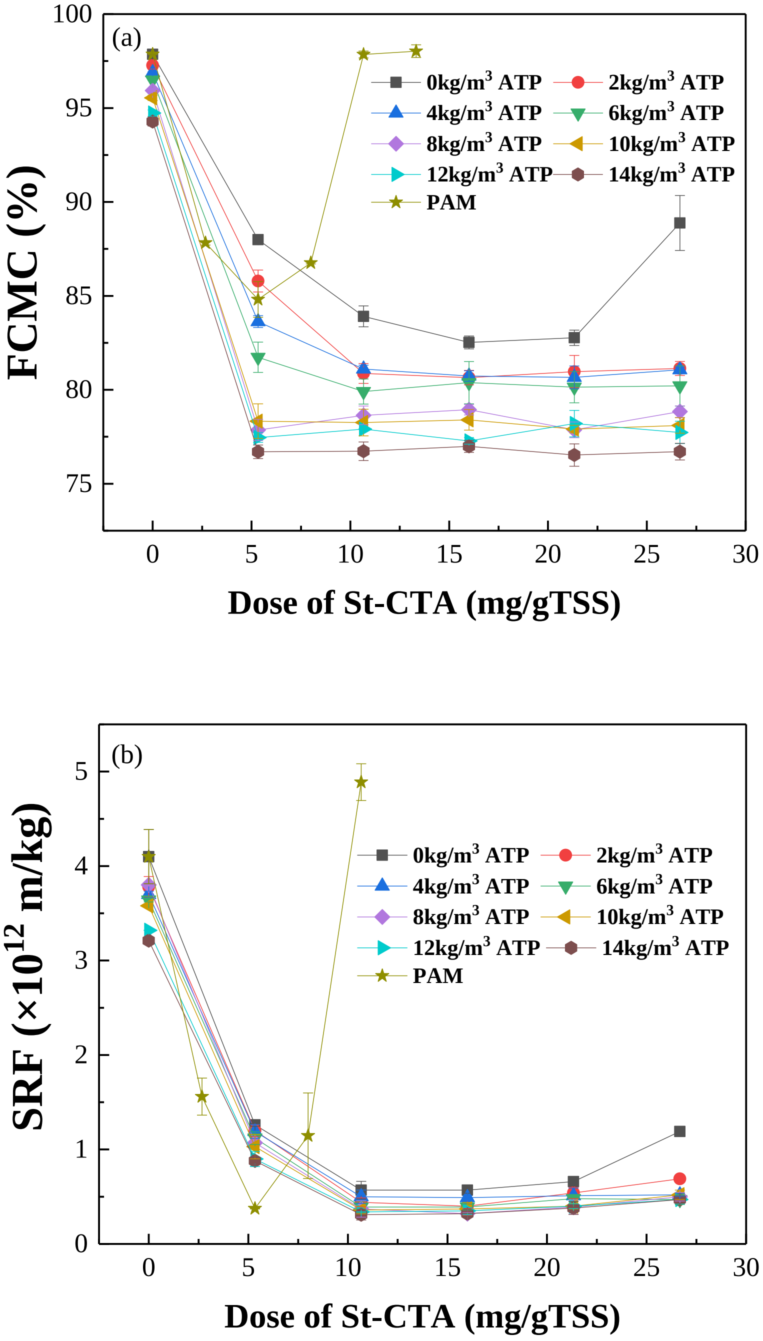

Figure S8 The sludge dewatering performance of St-CTA in conjunction with ATP by using different doses compared with PAM: (a) filter cake moisture content and (b) SRF.

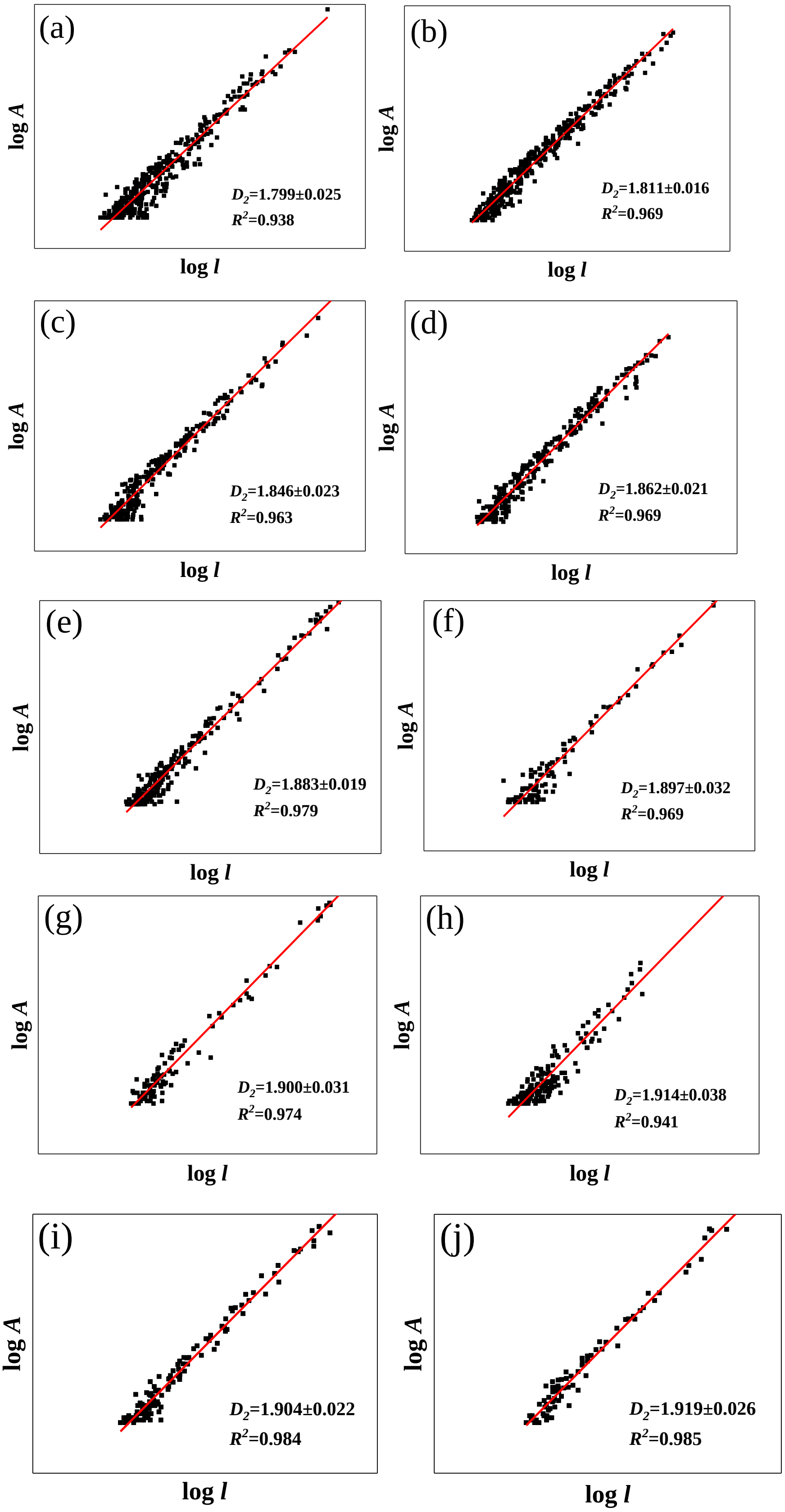
(a)
D_2=1.799±0.025
R^2=0.938
(b)
D_2=1.811±0.016
R^2=0.969
(c)
D_2=1.846±0.023
R^2=0.963
(d)
D_2=1.862±0.021
R^2=0.969
(e)
D_2=1.883±0.019
R^2=0.979
(f)
D_2=1.897±0.032
R^2=0.969
(g)
D_2=1.900±0.031
R^2=0.974
(h)
D_2=1.914±0.038
R^2=0.941
(i)
D_2=1.904±0.022
R^2=0.984
(j)
D_2=1.919±0.026
R^2=0.985
log A
log l

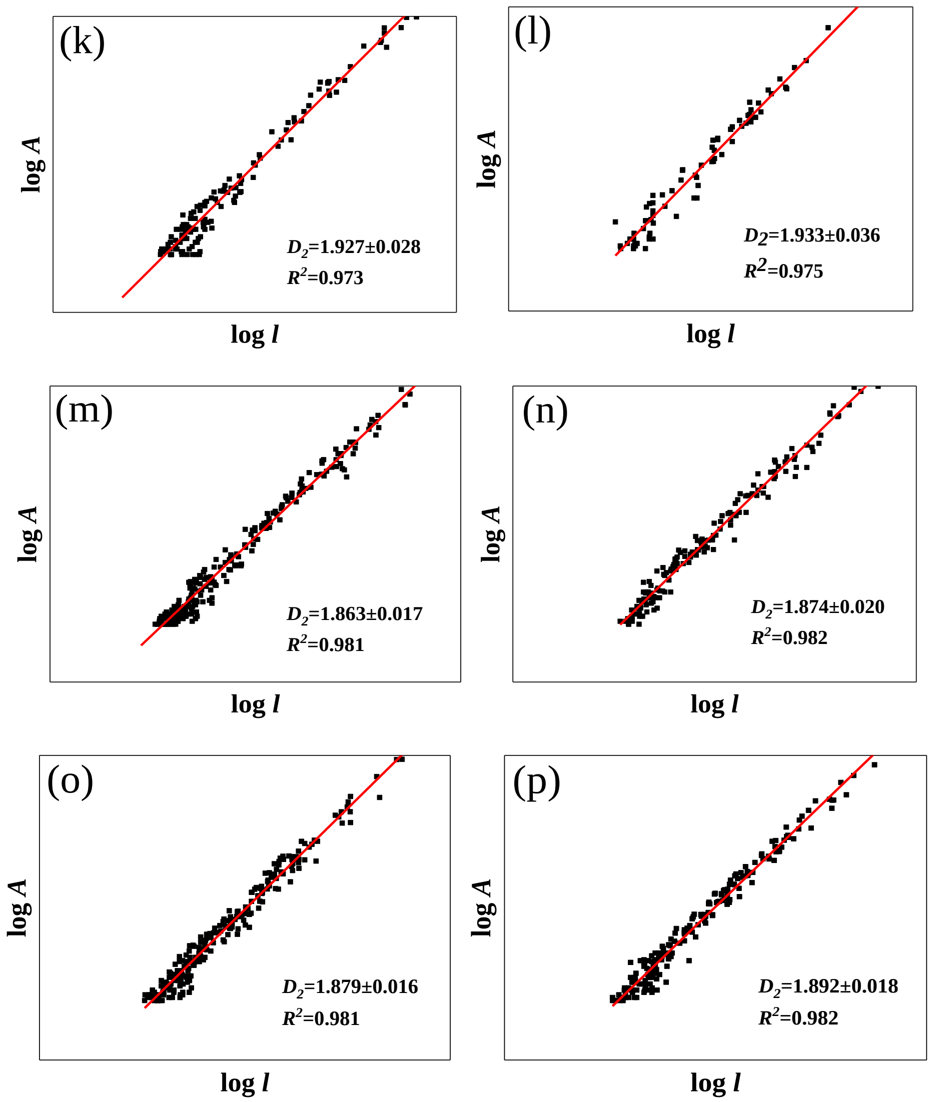

Figure S9 Image analysis of various sludge flocs conditioned by St-CTA in conjunction with ATP at various doses: (a) raw-sludge, (b) CS-ATP2, (c) CS-ATP3, (d)CS-ATP4, (e) CS-ATP5, (f) CS-ATP7, (g) CS-ATP8, (h) CS-ATP9, (i) CS-ATP10, (j) CS-ATP12, (k) CS-ATP13, (*l*) CS-ATP14, (m) CS-ATP15, (n) CS-ATP17, (o) CS-ATP18, and (p) CS-ATP19.

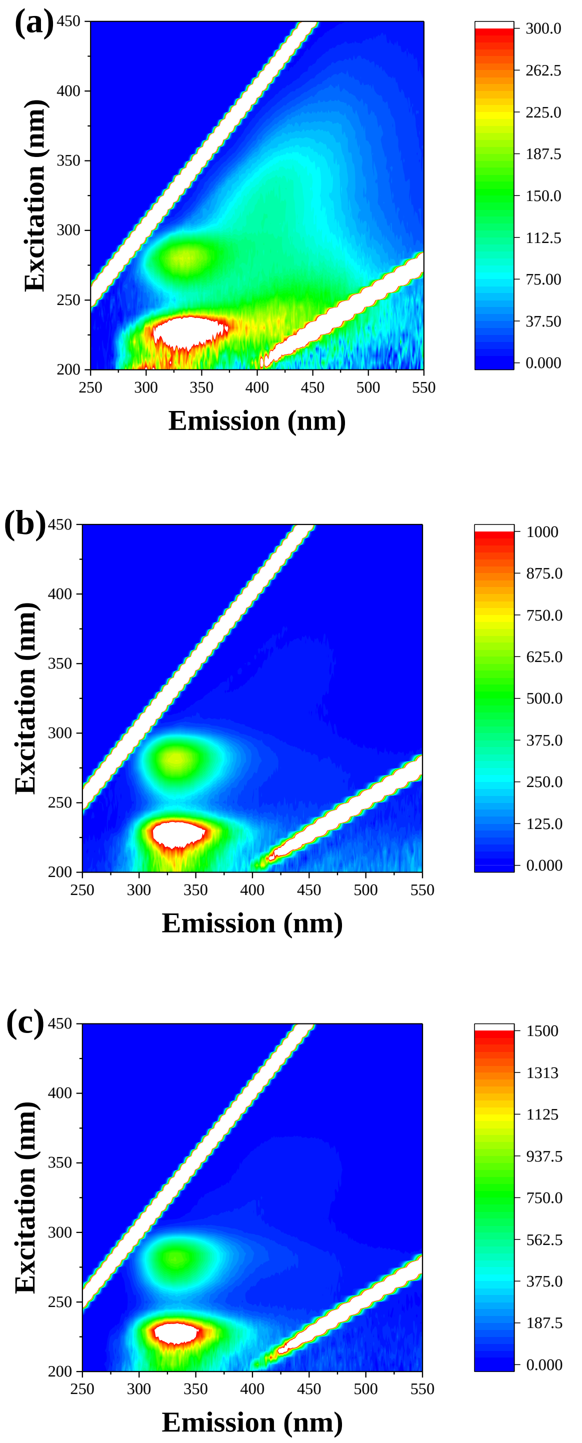

Figure S10 3D-EEM spectra of raw sludge: (a) S-EPS, (b) LB-EPS, and (c) TB-EPS fractions, respectively.

EPS fractions	Various CS-ATP samples			
	CS-ATP2	CS-ATP4	CS-ATP10	CS-ATP12
S-EPS	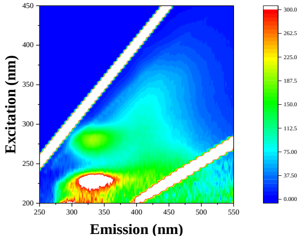	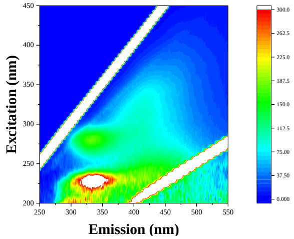	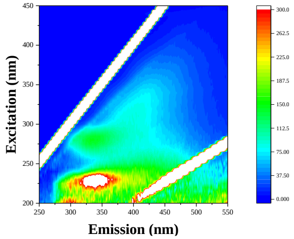	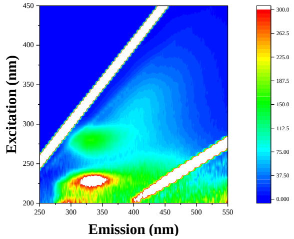
LB-EPS	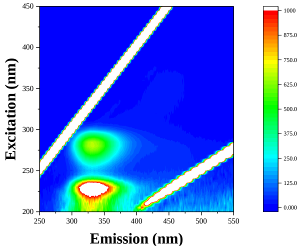	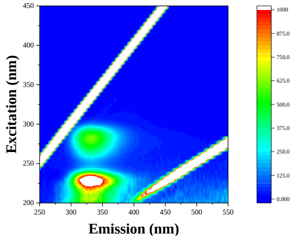	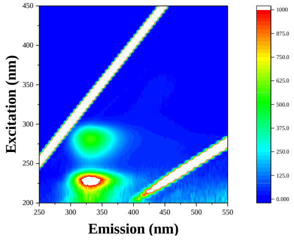	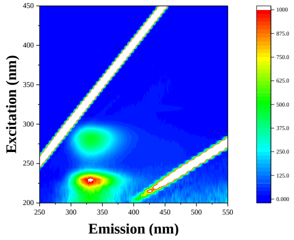
TB-EPS	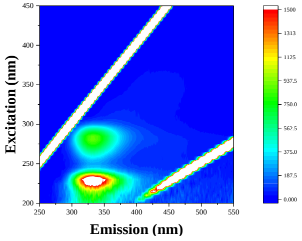	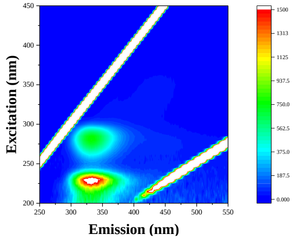	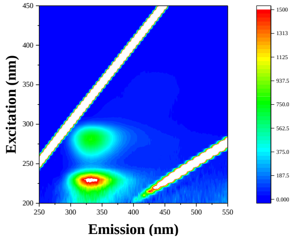	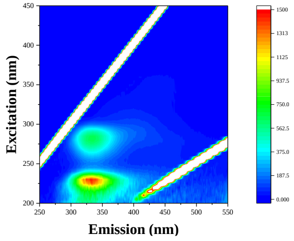

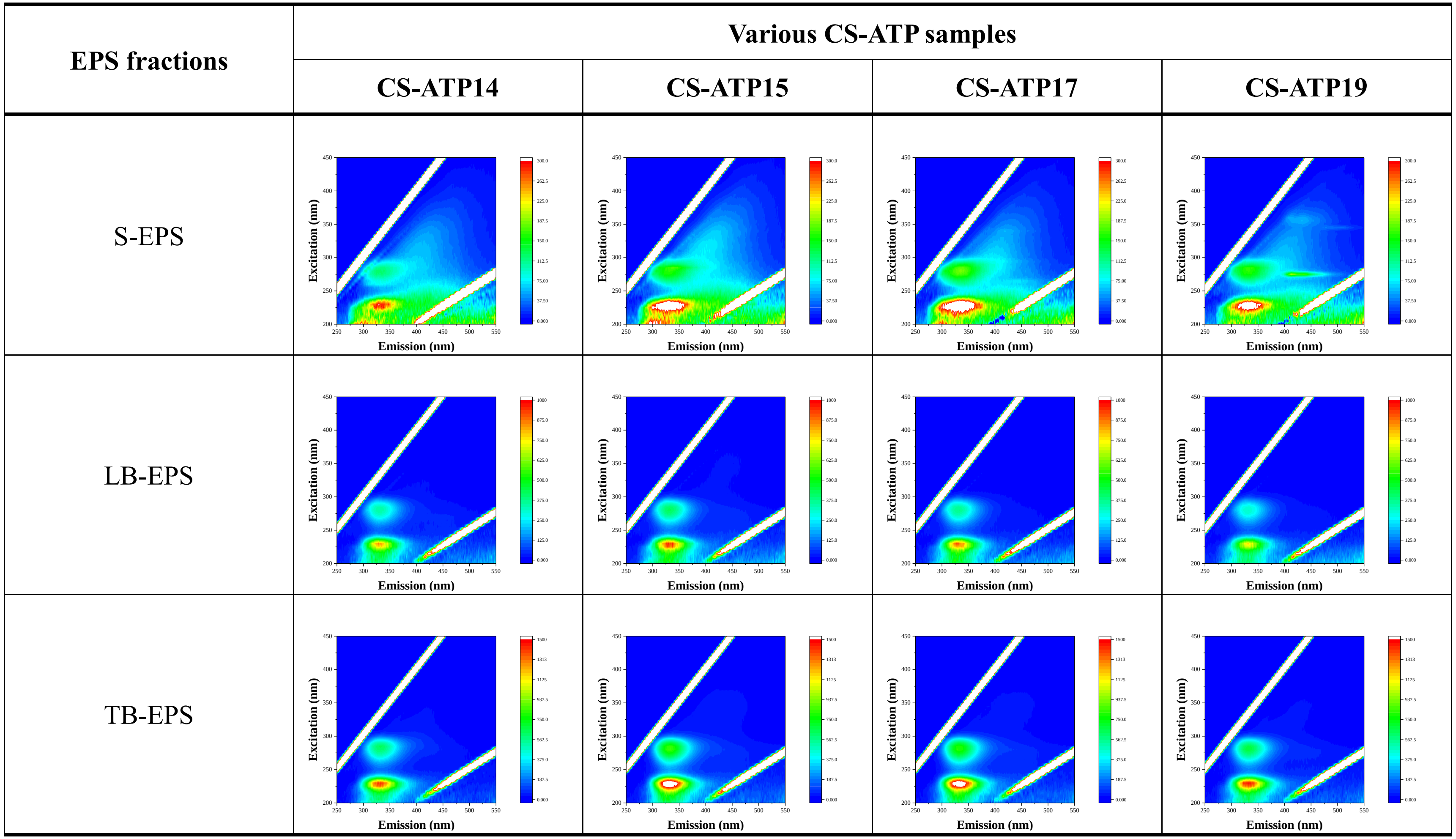

Figure S11 3D-EEM spectra of various EPS fractions in sludge conditioned by St-CTA in conjunction with ATP using different doses. (S-EPS samples were diluted by 10 times, while LB-EPS and TB-EPS samples were diluted by 50 times).
